# Alternating Hemiplegia of Childhood-Related Neural and Behavioural Phenotypes in Na^+^,K^+^-ATPase α3 Missense Mutant Mice

**DOI:** 10.1371/journal.pone.0060141

**Published:** 2013-03-20

**Authors:** Greer S. Kirshenbaum, Neil Dawson, Jonathan G. L. Mullins, Tom H. Johnston, Mark J. Drinkhill, Ian J. Edwards, Susan H. Fox, Judith A. Pratt, Jonathan M. Brotchie, John C. Roder, Steven J. Clapcote

**Affiliations:** 1 Samuel Lunenfeld Research Institute, Mount Sinai Hospital, Toronto, Ontario, Canada; 2 Institute of Medical Science, University of Toronto, Toronto, Ontario, Canada; 3 Strathclyde Institute of Pharmacy and Biomedical Sciences, University of Strathclyde, Glasgow, United Kingdom; 4 Institute of Life Science, College of Medicine, Swansea University, Swansea, United Kingdom; 5 Division of Brain, Imaging and Behaviour – Systems Neuroscience, Toronto Western Research Institute, Toronto, Ontario, Canada; 6 Division of Cardiovascular and Neuronal Remodelling, Leeds Institute for Genetics, Health and Therapeutics, University of Leeds, Leeds, United Kingdom; 7 School of Biomedical Sciences, University of Leeds, Leeds, United Kingdom; University of Edinburgh, United Kingdom

## Abstract

Missense mutations in *ATP1A3* encoding Na^+^,K^+^-ATPase α3 have been identified as the primary cause of alternating hemiplegia of childhood (AHC), a motor disorder with onset typically before the age of 6 months. Affected children tend to be of short stature and can also have epilepsy, ataxia and learning disability. The Na^+^,K^+^-ATPase has a well-known role in maintaining electrochemical gradients across cell membranes, but our understanding of how the mutations cause AHC is limited. *Myshkin* mutant mice carry an amino acid change (I810N) that affects the same position in Na^+^,K^+^-ATPase α3 as I810S found in AHC. Using molecular modelling, we show that the *Myshkin* and AHC mutations display similarly severe structural impacts on Na^+^,K^+^-ATPase α3, including upon the K^+^ pore and predicted K^+^ binding sites. Behavioural analysis of *Myshkin* mice revealed phenotypic abnormalities similar to symptoms of AHC, including motor dysfunction and cognitive impairment. 2-DG imaging of *Myshkin* mice identified compromised thalamocortical functioning that includes a deficit in frontal cortex functioning (hypofrontality), directly mirroring that reported in AHC, along with reduced thalamocortical functional connectivity. Our results thus provide validation for missense mutations in Na^+^,K^+^-ATPase α3 as a cause of AHC, and highlight *Myshkin* mice as a starting point for the exploration of disease mechanisms and novel treatments in AHC.

## Introduction

Alternating hemiplegia of childhood (AHC) is a rare but severe disease that is difficult to diagnose and even more challenging to treat. The disease has an estimated prevalence of one case per million births [1], but this is probably an underestimate caused by variability in clinical presentation, lack of awareness, and frequent misdiagnosis as epilepsy or cerebral palsy. Onset of AHC usually occurs before the age of 6 months, manifesting mainly as ocular movements and dystonic attacks or an episode of marked hypotonia [2]. Patients have bouts of hemiplegia or hemiparesis that last a few minutes to several days [1]. Other paroxysmal symptoms, such as tonic/dystonic spells or autonomic disturbances, including alterations in skin colour, temperature, and sweating, are also present, either concurrent with hemiplegia or in isolation. Attacks are often precipitated by triggers such as extremes of temperature, specific foods, physical activity, or exposure to water or changes in lighting [3]. Proven therapies for amelioration of episode frequency and duration are extremely limited, and the long-term impact of even the most frequently prescribed drug treatment, flunarizine, is unknown [3].

Between episodes, patients present with permanent neurological symptoms, including poor motor organizational skills, tremor, ataxia, involuntary abnormal movements, developmental delay and intellectual disability [3], [4]. The hemiplegic attacks in AHC are reported to be more severe during the first decade, after which they tend to be milder, whereas cognitive delay becomes more evident [2]. Among 92 patients who met clinical diagnostic criteria for AHC, the most frequent non-episodic symptoms were ataxia (96%) and cognitive impairment (100%), which result in severe disability as the disease advances [1], [3]. Children with AHC are also prone to a wide range of behavioural and psychiatric disorders, including impulsivity, attention deficit disorder, difficulties in acquiring speech, obsessionality, and short-temperedness [1]. None of the treatment interventions has a prominent effect on the non-episodic symptoms [4]. The most common finding of single-photon emission computed tomography (SPECT) and 2-Deoxy-d-glucose (2-DG) positron emission tomography (PET) is an interictal decrease in cerebral metabolism [3], [5]. AHC patients also tend to be of short stature and low weight, which may be related to difficulties in chewing and swallowing [1], [4].

Next-generation sequencing has identified 22 different *de novo* heterozygous missense mutations in *ATP1A3* gene encoding Na^+^,K^+^-ATPase α3 in 103 of 129 (80%) AHC patients studied [6], [7]. AHC patients with *ATP1A3* mutations were significantly more likely to have seizures (54% versus 29%) [7]. The α-subunit is the catalytic component of the Na^+^,K^+^-ATPase, which mediates the transport of sodium (against its gradient) from the cell cytosol to the extracellular fluid in exchange for potassium in the opposite direction (also against its gradient). In each catalytic cycle, three Na^+^ ions are actively extruded from the cell in exchange for two K^+^ ions per ATP hydrolyzed [8]. Neurones dedicate up to two-thirds of the energy of ATP hydrolysis just to maintain this active transport process [9]. Three α-subunit isoforms are expressed in mammalian brain: α1 in various cell types, α2 predominantly in astrocytes, and α3 exclusively in neurones [8].

Missense mutations in *ATP1A3* were previously identified as the primary cause of rapid-onset dystonia-parkinsonism (RDP; DYT12), a disorder characterised by abrupt onset of the permanent symptoms of dystonia with parkinsonism, often after a stressful event, typically in late adolescence or early adulthood [10], [11]. No mutations identified to date are shared between the two disorders, but three AHC-causing mutations affect amino acid positions also affected by RDP mutations (I274N/T, D801N/Y, D923Y/N). Nearly all identified AHC mutations affect regions in or near transmembrane (TM) domains of Na^+^,K^+^-ATPase α3, whereas the RDP mutations are more evenly distributed throughout the protein. *In vitro* analyses have shown that RDP mutations typically reduce Na^+^,K^+^-ATPase α3 protein expression [7], [10], whereas AHC mutations caused reductions of 75–90% in Na^+^,K^+^-ATPase activity without affecting the level of Na^+^,K^+^-ATPase α3 expression [7]. RDP mutations, but not AHC mutations, have been observed in asymptomatic carriers without identifiable motor symptoms [11]. It has been postulated that specific amino acid changes might explain the relatively mild and the late onset clinical phenotype of RDP compared with AHC [6].

Heterozygous amino acid change I810S that was found in a child with AHC affects the same position in Na^+^,K^+^-ATPase α3 as amino acid change I810N in heterozygous *Myshkin* (*Atp1a3^Myk^* ^/*+*^; *Myk*/+) mutant mice (Figure S1A) [7], [12]. I810N results in a normally expressed but inactive Na^+^,K^+^-ATPase α3 enzyme, which is consistent with the known effects of AHC mutations [7], [12]. Total Na^+^,K^+^-ATPase activity (α1 + α2 + α3) in the brains of *Myk*/+ mice is reduced by 36–42% [12], [13]. Consistent with the low weight and increased incidence of epilepsy and psychiatric disorders in AHC patients [1], [7], [14], *Myk*/+ mice exhibit an 18% reduction in body weight, neuronal hyperexcitability with occasional convulsions [12], and mania-related behaviour responsive to lithium and valproate [13]. When subjected to vestibular stress, *Myk*/+ mice exhibit transient tonic attacks and staggering movements that, in one-third of cases, develop into tonic-clonic seizures accompanied by epileptic discharges [12]. When bred to homozygosity, *Myk*/*Myk* pups appear grossly normal but die shortly after birth [12].

Herein, we report the use of molecular modelling of the structural effects on Na^+^,K^+^-ATPase α3 of the I810N *Myshkin* mutation in comparison with mutations known to cause AHC or RDP in human carriers. Following this analysis, we analyzed heterozygous I810N *Myk*/+ mice in several behavioural tests of motor and cognitive function to determine whether they model any of the permanent symptoms of AHC. Finally, to provide greater insight into the nature of Na^+^,K^+^-ATPase α3 missense mutation-induced alterations in brain functioning – which may have translational relevance to AHC – we undertook 2-DG autoradiographic imaging of I810N *Myk*/+ mouse brains.

## Materials and Methods

### Ethics statement

Full details of the study were approved after review by the University of Leeds Ethical Review Committee and the humane care and use of mice in this study was carried out under the authority of the appropriate UK Home Office Project Licence (40/3447) in accordance with the requirements of the Animals (Scientific Procedures) Act 1986.

### Structural modelling of Na^+^,K^+^-ATPase α3 mutations

Wild-type and mutant forms of mouse Na^+^,K^+^-ATPase α3 were modelled by 85% homology with the recently reported crystal structure of Na^+^,K^+^-ATPase α3 with bound potassium and ouabain (PDB: 2ZXE; previously 3A3Y) from spiny dogfish (*Squalus acanthias*) [15], using a homology modelling pipeline built with the Biskit structural bioinformatics platform [16]. The mutants included the I810N *Myshkin* mutation, four AHC mutations (I274N, D801N, I810S, D923Y) and three RDP mutations (I274T, D801Y, D923N) (Table 1). Our pipeline workflow incorporates the NCBI tools platform [17], including the BLAST program for similarity searching of sequence databases. T-COFFEE [18] was used for alignment of the test sequence with the template, followed by iterations of the MODELLER-9.11 program (September 2012 release) [19] to calculate a structural model. The K^+^ binding sites were predicted by superposition of the mouse Na^+^,K^+^-ATPase α3 models upon the 2ZXE structure using the Chimera program [20], which was also used for the viewing of models and generation of images. It is not currently possible to predict the position of the Na^+^ binding sites due to the lack of a determined Na^+^ bound structure.

**Table 1:** Na^+^,K^+^-ATPase α3 mutations selected for structural modelling.

Mutation	Species	Phenotype	Na^+^,K^+^-ATPase α3 expression				Na^+^,K^+^-ATPase α3 activity	
			HeLa cells^[7]^	COS-7 cells^[7]^	HEK293T cells^[10]^	Mouse brain^[12]^	COS-7 cells^[7]^	Mouse brain^[12]^
I274N	Human	AHC	n.d.	n.d.	n.d.	n.d.	n.d.	n.d.
I274T	Human	RDP	↓	↓	↓	n.d.	n.d.	n.d.
D801N	Human	AHC	=	=	n.d.	n.d.	↓	n.d.
D801Y	Human	RDP	=	=	↓	n.d.	↓	n.d.
I810N	Mouse	*Myshkin*	n.d.	n.d.	n.d.	=	n.d.	↓
I810S	Human	AHC	n.d.	n.d.	n.d.	n.d.	n.d.	n.d.
D923N	Human	RDP	=	=	n.d.	n.d.	↓	n.d.
D923Y	Human	AHC	n.d.	n.d.	n.d.	n.d.	n.d.	n.d.

↓ lower than wild-type;  =  no significant difference; n.d. not determined.

### Mice

The *Myshkin* mouse line has been previously described and was backcrossed 20 generations to the seizure-resistant C57BL/6NCr strain (NCI-Frederick) [12], [13]; *Myk*/+ mice at N_20_ C57BL/6NCr were previously reported not to show stress-induced seizure activity during electrocorticography [13]. Mice used in the present study were bred from C57BL/6NCrl females (Charles River) and *Myk*/+ males, and were genotyped by the presence of an *Eco*O109I (New England BioLabs) restriction site using polymerase chain reaction (PCR) primers F, 5′-CTG CCG GAA ATA CAA TAC TGA-3′ and R, 5′-ATA AAT ACC CCA CCA CTG AGC-3′. Wild-type littermates were used as controls for all experiments. At 4 weeks of age, pups of mixed genotypes were weaned and housed by sex in groups of three to five. Mice were housed in filtered cages containing corn cob bedding (Bed-O'-Cobs, Andersons), nesting material (Nestlets, Ancare) and ad libitum sterile food (2018 Teklad Rodent Diet) and water. Housing conditions were maintained at 21±1°C and 50–60% humidity under a 12∶12 h light-dark cycle (lights on: 0700–1900 hours). *Atp1a3^Myk^* ^/*+*^ mice are available from the Canadian Mouse Mutant Repository (http://www.cmmr.ca/mutants_samples/index.html).

### Behavioural tests

Behavioural tests were conducted on *Myk*/+ and +/+ littermates at 8–12 weeks of age. Male and female mice were included in experiments in balanced numbers, except for the pawprint analysis, which included only females because the males were required to propagate the *Myshkin* line in a new animal facility. Subjects were handled daily for 5 min/day for 7 days prior to behavioural testing. Testing was conducted during the light phase (0900–1700 hours). Prior to experiments, subjects were left undisturbed in the testing environment for 30 min to allow for acclimation. A solution of 70% ethanol or Clidox-S was used to clean surfaces and equipment between subjects.

#### Gait analysis

Gait analysis was conducted by coating the fore and hind paws with two colours of non-toxic paint, and allowing mice to walk along a narrow, paper-covered runway. Gait was assessed by analyzing the resulting pawprint patterns, as described previously [21], except that measurements were expressed per cm of trunk (defined as the distance between the forelimbs and hindlimbs) to account for the smaller body size of *Myk*/+ mice [12].

#### Balance beam

Mice were given five training trials on an 80-cm long, 20-mm wide beam elevated 50 cm above a padded base, as described previously [21]. A 60-W lamp at the start platform served as an aversive stimulus, whereas the opposite end of the beam entered a darkened escape box baited with food pellets. The number of foot slips and traversal time were measured as mice traversed the beam in a test trial 24 hours after training.

#### Tail suspension

Mice were suspended by their tails 30 cm above a surface for 30-s and observed for hindlimb clasping, a stereotyped behavioural phenotype indicative of neurological dysfunction. A mouse was allocated a score  = 1 for abnormal hindlimb movement and score  = 0 in the absence of any abnormal movement in each 10-s epoch, allowing a maximum score of 3. Abnormal movement was defined as the retraction of either or both hindlimbs into the body and toward the midline. Wild-type mice often splay their hindlimbs out when suspended.

#### Grip strength

The grip strength of all four limbs and the forelimbs alone was measured using a digital grip strength gauge (Grip Strength System, San Diego Instruments), as described previously [21]. The mouse was held by the tail and lowered onto a wire mesh grid until it could easily grip the grid with all four limbs or the forelimbs. The tail was then steadily and horizontally pulled away from the grid until the mouse released its grip. The maximal force (g) required to relieve the grip was recorded. Each mouse was subjected to three trials separated by 5-min intervals. The average score of three trials for each mouse was reported.

#### Other motor behavioural tests

Tremor was measured with a commercial tremor monitor (San Diego Instruments) according to the manufacturer's instructions. Briefly, mice were placed in the detection tube and allowed 5 min to habituate. After habituation, the tremor amplitude was measured for each mouse for 256 s. The accelerating rotarod test was conducted as described previously [21], except that mice were given three trials each day for five consecutive days. Acoustic startle response was measured as previously described [22]. Data were analysed using one-way analysis of variance (ANOVA).

#### Fear conditioning

Experiments were conducted in a fear conditioning chamber (MED Associates; 25 cm height ×30 cm width ×25 cm length), and automated fear conditioning software was used to score behaviour (FreezeFrame 1.6e, Actimetrics). For the training phase, mice were placed in the chamber for 2 min followed by a 30-s auditory tone (3600 Hz, 95 dB). Mice received a continuous scrambled foot shock of either 1.0 mA (Cohort 1) or 0.75 mA (Cohort 2) during the last 2 s of tone and remained in the chamber for an additional 30 s before being returned to their home cage. 24 h following training, mice were returned to the fear conditioning chamber to evaluate their contextual fear memory. Freezing to the context was recorded for 3 min, and then mice were returned to their home cage. 26 h following training, the chamber was altered to evaluate freezing to the auditory tone. The grid was covered with a smooth white Perspex sheet, the walls were altered by inserting a white Perspex triangle, a 1% acetic acid odour was applied and the lights in the room were switched off. Subjects were placed in a chamber for 3 min to explore the new environment, followed by a 3 min presentation of the auditory tone while freezing was recorded. Data were analysed using one-way ANOVA and Student's two-sample *t* test.

#### Conditioned taste aversion

The CTA procedure was adapted from a previously published method [23]. For the 7-day duration of the CTA procedure, mice were group housed and water-deprived with free access to food. During testing sessions that occurred once every 24 hours, mice were placed individually into separate cages that contained drinking bottles. During a 5-day habituation period, mice were trained to drink their daily water ration from two bottles containing room temperature autoclaved water within 30 min (Day 1, 120 min access to water; Days 2 and 3, 60 min access; Days 4 and 5, 30 min access). On the conditioning day, a novel taste (the CS; 0.1% w/v saccharin sodium salt) was paired with the malaise-inducing agent LiCl (0.3 M, 2% body weight, i.p.). Placement of the CS solution bottle (left vs. right) was counterbalanced across mice. The CS fluid was presented for 30 min and, 40 min later, mice were treated with LiCl or a similar volume of saline. Testing occurred 24 h later, when two bottles (one bottle containing water and the other containing the CS fluid) were presented for 30 min. The intake of each fluid was measured. The degree of CTA learning was determined for each mouse by a CS consumption score: the amount of CS solution consumed divided by the total amount of fluid (CS + water) consumed. Data were analysed using two-way ANOVA.

### Assessment of [^14^C]-2-deoxyglucose utilization

An injection of 80 µCi/kg [^14^C]-2-deoxyglucose (specific activity 56 mCi/mmol; GE Healthcare) in sterile saline was administered interperitoneally at a volume of 10 ml/kg to *Myk*/+ (*n = *10) and +/+ (*n = *8) mice, as described previously [24], [25]. Forty-five minutes after 2-DG administration, mice were decapitated and the brains rapidly removed, snap-frozen in isopentane cooled to −42°C, and stored at −70°C until preparation of sections for autoradiographic analysis.

Tissue was sectioned in the coronal plane using a cryostat (20 µm) throughout the whole brain. Sections were thaw-mounted onto slides and immediately placed on a slide heater at 65°C to ensure rapid desiccation and prevent lateral diffusion of 2-DG. Slides along with ^14^C standards were then sealed within autoradiographic cassettes and opposed to beta-sensitive film (Kodak) for 21 days at −80°C. Films were developed using an automatic processor (Konica) prior to analysis using MCID 6.0 Elite image analysis system software (MCID). Densiometric analysis of each section was carried out whereby a reference curve of [^14^C] (nCi/g) versus optical density was calculated from the coexposed beta-emitting [^14^C] microscale standards and used to quantify the density signal for each brain region into its [^14^C] concentration. Background intensity was subtracted from each reading. Values were obtained from 45 regions of interest (RoI) throughout the brain (including the cerebellum) with reference to a stereotaxic mouse brain atlas [26]. Regional glucose metabolism was estimated as the ^14^C concentration in each RoI relative to the whole-brain average ^14^C level (defined as the average concentration of ^14^C across all analysed regions) in the same mouse, consistent with published reports using the same semi-quantitative 2-DG approach [25]. Significant differences in regional metabolism between *Myk*/+ and +/+ mice were assessed using Welch's *t*-test, which was used due to unequal variances in the samples. In line with previous protocols [25], the functional connectivity of defined “seed” RoI was determined in *Myk*/+ and +/+ mice using the partial least squares regression algorithm (PLSR). PLSR analysis was completed using the PLS package in R [27], [28]. “Seed” RoI were selected on the basis of a significant difference in overt local cerebral glucose utilization (LCGU) between *Myk*/*+* and +/+ mice. In this way, *Myk*/*+*-induced alterations in the functional connectivity of the frontal cortex (FCTX), three thalamic nuclei (ventral anterior thalamic nucleus [VAthal], ventromedial thalamic nucleus [VMthal], ventral posteromedial thalamic nucleus [VPMthal]), two subfields of the periaqueductal grey (dorsomedial [DMPAG] and rostral [RPAG]), and the caudal motor cortex (CMCTX) were considered. The functional connectivity of each ‘‘seed’’ region (dependent variable in the PLSR model) to all of the other RoI measured (explanatory variables; 44 brain regions in each analysis) was considered in terms of the variable importance to the projections (VIP) statistic. Within each experimental group, and for each ‘‘seed’’ region, a significant functional connection between brain regions was considered to exist if the 95% confidence interval (CI) for the VIP statistic exceeded 0.8, because this threshold denotes a considerable contribution of an explanatory variable to the dependent variable in PLSR models. The standard deviation (SD) and CI for the VIP statistic were estimated by jackknifing. The significance of *Myk*/*+* induced alterations in functional connectivity (the VIP statistic) was analyzed by comparison to the real difference in the VIP statistic between experimental groups relative to that in 1,000 random permutations of the raw data. Significance was set at *P<*0.05 throughout.

### Measurement of arterial blood pressure

Arterial blood pressure (mean, systolic, and diastolic) and heart rate were measured by radiotelemetry, as previously described [29]. The probe catheter was advanced into the ascending aorta via the left carotid artery. Twenty-four-hour recordings were obtained by sampling for 2 min every 15 min. For Millar catheter analysis, a pressure–volume catheter was inserted into the left ventricle via the right carotid artery. Data were analysed using Student's *t* test.

## Results

### *Myshkin* and AHC mutations have greater impacts than RDP mutations on Na^+^,K^+^-ATPase α3 structure

To investigate a potential structural rationale for the disease phenotype pertaining to each missense mutation of Na^+^,K^+^-ATPase α3, we undertook comparative molecular modelling of the I810N *Myshkin* mutation along with the AHC mutations and RDP mutations affecting the same positions (Table 1). All the mutations studied affect transmembrane helices bordering the K^+^ pore (Figure S1B). Mechanisms for deleterious effects can be readily postulated for all the *Myshkin*, AHC and RDP mutations, though there is a consistent pattern of the AHC mutations and *Myshkin* displaying more severe structural impacts than the RDP mutations affecting the same positions, notably upon the K^+^ pore and predicted K^+^ binding sites. It is likely that there are significant impacts upon Na^+^ binding and transport too, but these cannot currently be definitively predicted by molecular modelling, due to the lack of a Na^+^ bound Na^+^,K^+^-ATPase structure.

**Figure 1:**
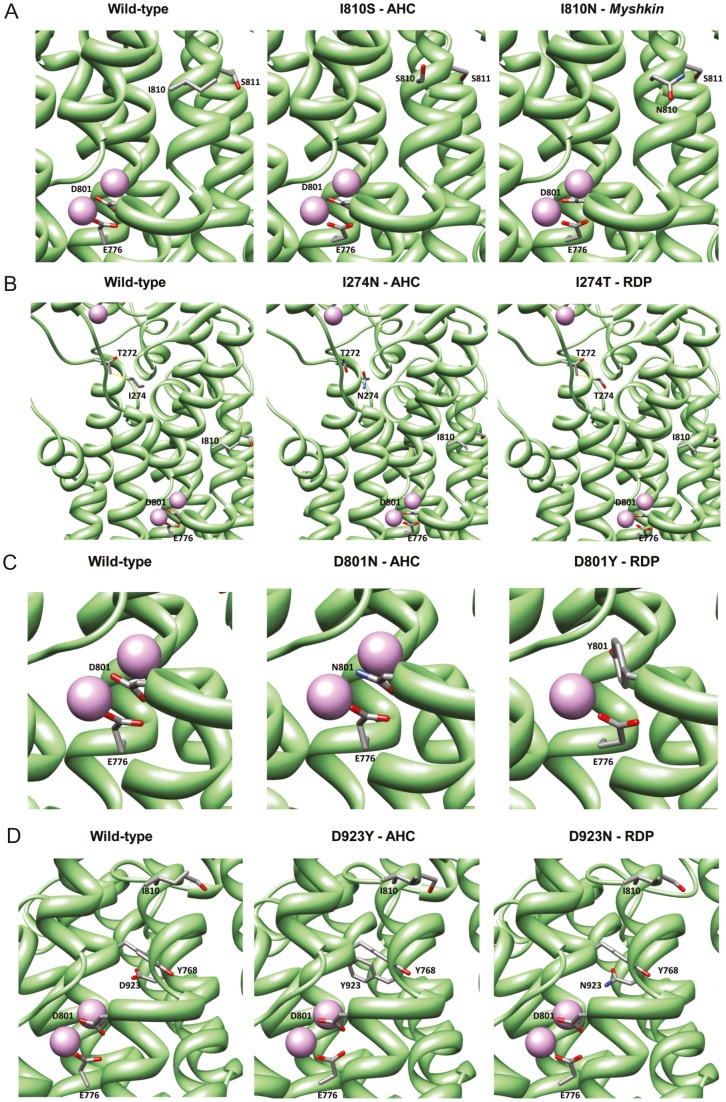
Structural modelling of Na^+^,K^+^-ATPase α3 mutations. (A) Na^+^,K^+^-ATPase α3 wild-type (left), the I810S mutant (AHC; centre) and the I810N mutant (*Myshkin*; right). (B) Na^+^,K^+^-ATPase α3 wild-type (left), the I274N mutant (AHC; centre) and the I274T mutant (RDP; right). Side chain contact between Δ272 and Δ274 at the cytoplasmic end of the K^+^ pore is shown in yellow for the wild-type protein and the I274T mutant, but this contact is lost in the I274N mutant. (C) Na^+^,K^+^-ATPase α3 wild-type (left), the D801N mutant (AHC; centre) and D801Y mutant (RDP; right). In the D801N mutant, the electrostatic interaction at Δ801 with both K^+^ ions is lost due to replacement of terminal oxygen with nitrogen, resulting in the obstruction of the K^+^ pore, likely to markedly affect conductance rates through the pore, while the interaction of K^+^2 with E776 is maintained. In the D801Y mutant, there is a predicted loss of interaction of K^+^2 with E776. (D) Na^+^,K^+^-ATPase α3 wild-type (left), the D923Y mutant (AHC; centre) and the D923N mutant (RDP; right).

The AHC mutation, I810S, imparts similarly severe structural consequences upon pore architecture as the *Myshkin* mutation, I810N, by substitution of a hydrophobic residue bordering the pore with a polar side chain, affecting the routing of K^+^ ions. The I810S substitution leads to the loss of interaction of E776 with its K^+^ ion and the introduction of an oxygen end group to the vicinity of the pore at S810. The I810N substitution also disrupts the interaction of E776 with its K^+^ ion, and introduces a polar end group to the pore (Figure 1A).

The AHC I274N substitution brings about loss of interaction of E776 with its K^+^ ion and introduction of a polar side chain into the K^+^ pore at N274. There is also a loss of side chain contact between Δ272 and Δ274 at the cytoplasmic end of the K^+^ pore, compared to the wild-type. With the RDP I274T mutation, a relatively hydrophobic side chain is incorporated and a similar Δ272-Δ274 contact to wild-type is found (Figure 1B).

In the wild-type protein, D801 is located directly between the predicted binding sites for the two K^+^ ions at the centre of the transmembrane domain of the protein. E776 interacts with one of the K^+^ ions (1.95Å). The profound impact of the substitution of D801 by the very similar asparagine (N) residue is due to the most common AHC mutation, D801N [6], [7], directly affecting the main K^+^ binding site of the protein. Interestingly, the substitution of D801 by tyrosine (Y), a more conspicuous substitution in terms of residue physicochemical properties and size difference, might result in a lesser impact on K^+^ conductance than D801N. Although the RDP D801Y mutation drastically affects binding at one of the K^+^ binding sites, passage through the pore remains unaffected. Y801 is predicted to occupy the position of one of the K^+^ ions, preventing binding there, while the passage of the other K^+^ ion is unaffected, leaving the K^+^ pore less obstructed than for D801N. In contrast, D801N introduces the positive dipole of the terminal amino group directly between the two K^+^ ions at the primary binding site, potentially repelling binding events and passage of both ions (Figure 1C).

Finally, the AHC D923Y mutation directly leads to a loss of negative charge bordering the K^+^ pore, along with dramatic spatial conflicts between Y923 and Y768 (projected overlap 0.7Å), with a significant impact on interhelical packing of TM8 and TM5 likely, and subsequently upon the orientation of TM5 around the K^+^ pore. With the RDP D923N mutation, there is also replacement of the negative charge of aspartic acid (D) bordering the K^+^ pore but with a highly polar group of the very similar asparagine (N), with highly similar orientation too. There is consequently no effect on interhelical packing of TM8 and TM5 in the case of D923N (Figure 1D).

### Motor dysfunction in Myk/+ mice

**Figure 2:**
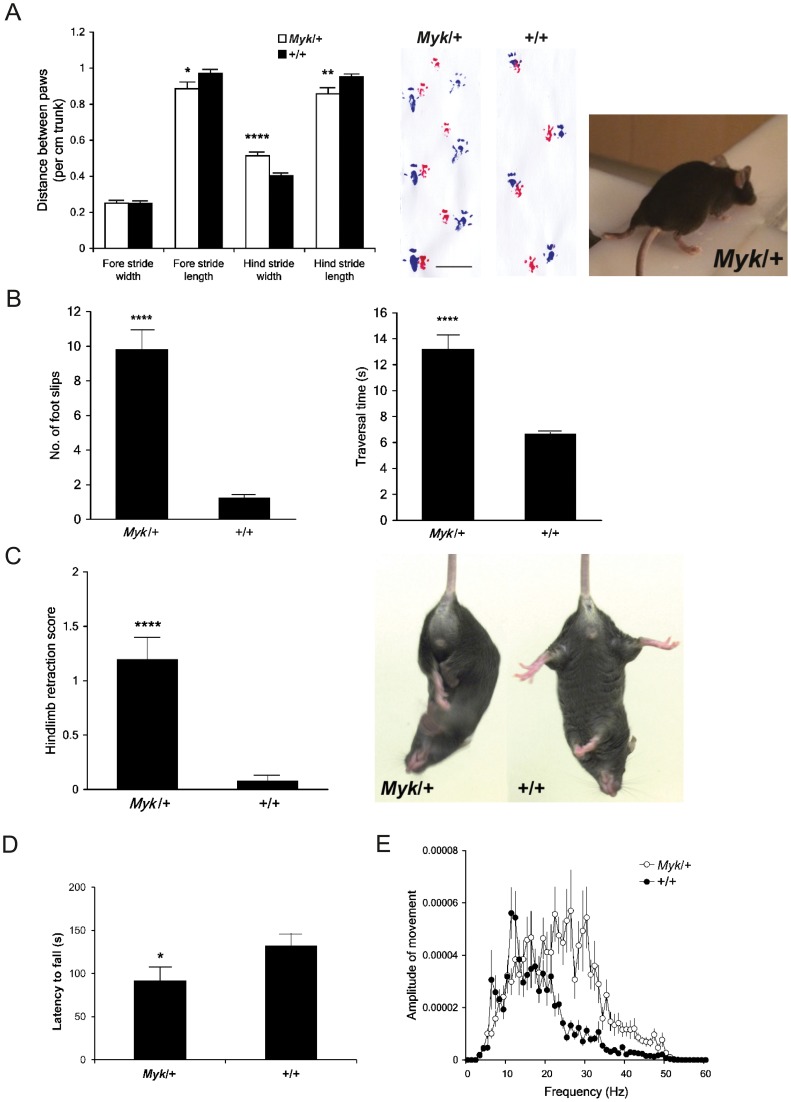
Motor dysfunction in *Myk*/+ mice. (A) Gait analysis. *Left panel*: Mean fore stride and hind stride distance (± SEM) per cm trunk of *Myk*/+ (*n = *14) and +/+ (*n = *17) female mice. There were significant main effects of genotype on fore stride length (*F*_1,30_ = 5.59, *P = *0.025), hind stride length (*F*_1,30_ = 8.09, *P = *0.008) and hind stride width (*F*_1,30_ = 24.44, *P = *0.0001) (left panel). *Middle panel*: Typical examples of forepaw (red) and hindpaw (blue) placement of *Myk*/+ and +/+ mice are shown. Scale bar  = 2 cm. *Right panel*: *Myk*/+ mouse showing splayed hindlimbs. (B) Balance beam. Mean number of foot slips (left panel) and traversal time (right panel) (± SEM) of *Myk*/+ (*n = *26) and +/+ (*n = *45) mice when traversing a narrow beam 24 hours after training. There were significant main effects of genotype on number of foot slips (*F*_1,70_ = 99.46, *P = *0.0001) and traversal time (*F*_1,70_ = 43.38, *P = *0.0001). (C) Tail suspension. Mean hindlimb retraction score (± SEM) of *Myk*/+ (*n = *26) and +/+ (*n = *26) mice suspended by the tail for 30 s. There was a significant main effect of genotype (*F*_1,51_ = 29.00, *P = *0.0001). Hindlimb retraction is defined as the movement of one of both hindlimbs into the central body axis (photograph). (D) Accelerating rotarod. Mean latency (± SEM) of *Myk*/+ (*n = *18) and +/+ (*n = *21) mice to fall from a rotating rod over three training trials. There were significant main effects of sex (*F*_1,38_ = 9.94, *P = *0.003) and genotype (*F*_1,38_ = 6.09, *P = *0.019), but not genotype x sex interaction (*F*_1,38_ = 0.91, *P = *0.346), females performing better than males regardless of genotype. (E) Tremor. Mean amplitude of displacement (± SEM) of *Myk*/+ (*n = *36) and +/+ (*n = *52) mice across a spectrum of frequencies. There was a significant main effect of genotype on frequency at the maximal amplitude (*F*_1,87_ = 57.1, *P = *0.0001). *P<0.05; **P<0.01; ****P<0.0001 versus +/+ mice.

*Myk*/+ mice of both sexes exhibit an unsteady, tremorous gait with occasional splaying of the hindlimbs from weaning (separation from the dam) at about 4 weeks of age (Figure 2A; Video S1, S2). Given the finding that the *Myshkin* mutation, I810N, imparts similarly severe structural consequences upon Na^+^,K^+^-ATPase α3 pore architecture as the AHC mutation, I810S, we proceeded to quantify the motor phenotype of *Myk*/+ mice in several behavioural tests.

Before conducting gait analysis, we compared the trunk length (defined as the distance between the forelimbs and hindlimbs) of *Myk*/+ and +/+ female mice as a measure of body size, since *Myk*/+ mice exhibit an 18% reduction in body weight [12]. *Myk*/+ mice had a significantly shorter trunk than +/+ mice (*Myk*/+ 5.61±0.19 cm versus +/+ 6.85±0.14 cm; *F*_1,30_ = 28.42, *P = *0.0001; Figure S2A). Gait analysis with adjustment for the genotypic difference in body size showed that, proportionately, *Myk*/+ mice walk with shorter strides and a broader base of the hindlimbs than their larger +/+ littermates (Figure 2A; unadjusted data in Figure S2B–F).

In the balance beam task, *Myk*/+ mice performed poorly and exhibited severe tremors and ataxic movement when walking across the beam. Some *Myk*/+ mice exhibited ‘hindlimb dragging’ at the start of training: their thorax and abdomen were flattened against the upper surface of the beam, their hindlimbs and tail were laterally wrapped around the beam, and their forelimbs were used to drag themselves along. In a trial 24 hours after training, the number of foot slips and the latency to traverse the beam were both significantly increased in *Myk*/+ mice compared with +/+ littermates (Figure 2B). In response to tail suspension, *Myk*/+ mice displayed frequent paroxysmal bouts of hindlimb clasping and trunk flexion within the 30 s period of observation, indicative of neurological disease of a generalized nature; 18 of 26 *Myk*/+ mice (69%) displayed at least one bout of hindlimb retraction compared with 2 of 26 of +/+ mice (8%) (Figure 2C). In the accelerating rotarod test, mice were given three trials each day for five consecutive days. On the first day, when subjects were naïve to the task, *Myk*/+ mice had difficulty remaining on the rotating rod and had a significantly shorter latency to fall than +/+ mice (Figure 2D). However, on the four subsequent days, we found no genotypic differences in performance on the accelerating rotarod (Figure S3A). Quantitative tremor monitoring revealed that the tremor of *Myk*/+ mice has a maximal amplitude at 22.67±1.079 Hz, which contrasts to the normal physiologic tremor of 13.52±0.68 Hz detected in +/+ mice (Figure 2E).

To test whether the motor dysfunction of *Myk*/+ mice was related to muscle weakness, we measured grip strength. There was no genotypic difference in forelimb strength among either sex, or strength of all four limbs among females. However, *Myk*/+ males required 11.7±2.6% less force to release all four paws from a wire grid (Figure S3B), but this difference was not maintained when grip strength was expressed per g of body weight (*Myk*/+ 15.01±0.37g versus +/+ 14.17±0.38g; *F*_1,49_ = 2.36, *P = *0.131). Grip strength of forelimbs and all four limbs showed a significant positive correlation with body weight in both genotypes (Figure S3C, D).

### Cognitive impairment in *Myk*/+ mice

**Figure 3:**
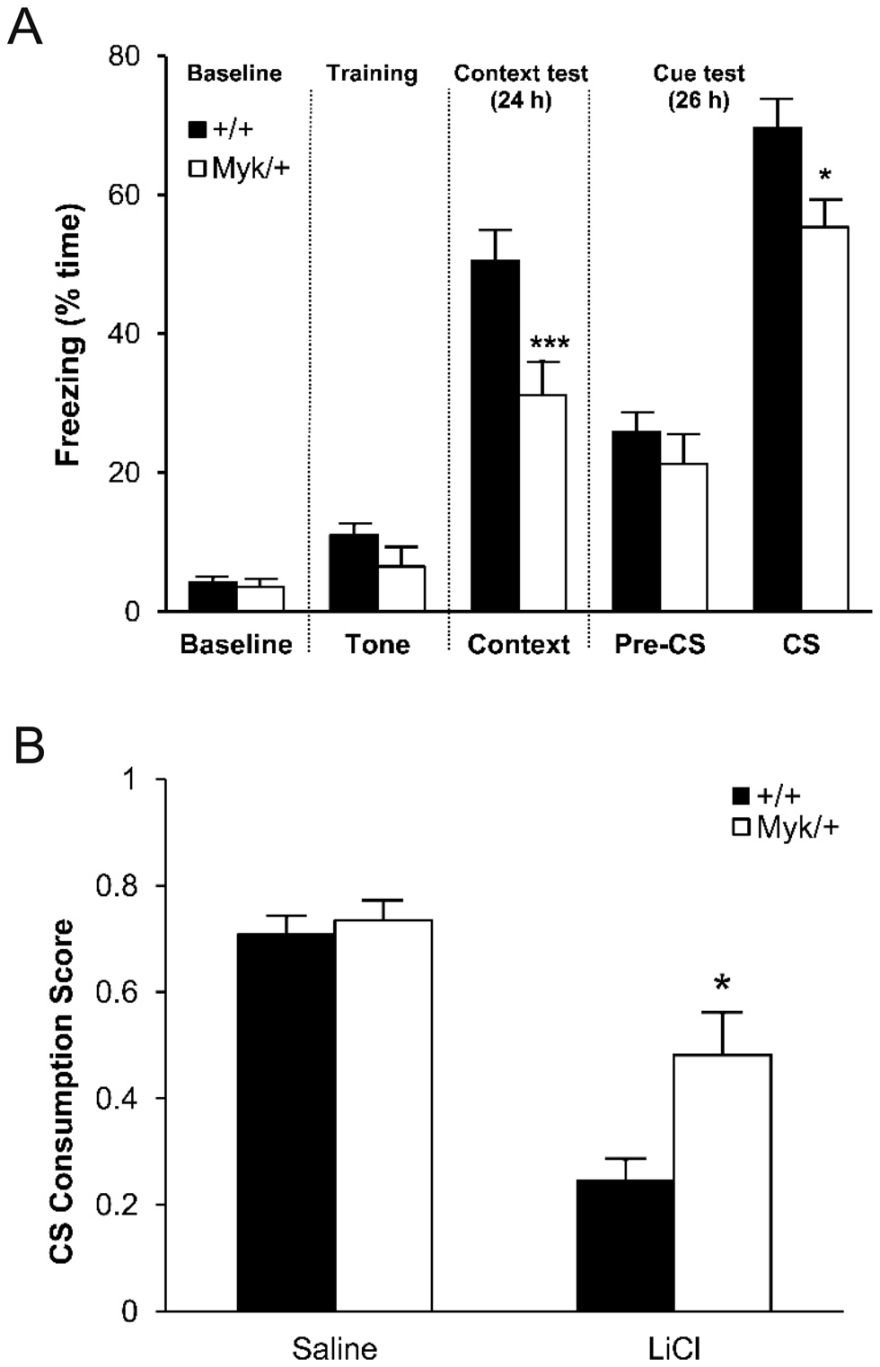
Cognitive impairment in *Myk*/+ mice. (A) Fear conditioning with 1.0-mA footshock. Mean freezing levels (± SEM) of *Myk*/+ (*n = *24) and +/+ (*n = *25) mice at baseline, during training, and in the contextual and cued conditioning tests. There were significant main effects of genotype on freezing in the context test (*F*_1,48_ = 8.52, *P = *0.005) and in the cue test during presentation of the auditory tone (CS; *F*_1,48_ = 6.20, *P = *0.016). (B) Conditioned taste aversion. Mean (± SEM) CS consumption scores (intake of saccharin/total fluid) 24 h following pairing with LiCl or saline treatment in *Myk*/+ and +/+ mice. There were significant main effects of genotype (*F*_1,47_ = 6.51, *P = *0.014), treatment (*F*_1,47_ = 48.12, *P = *0.0001), and genotype x treatment interaction (*F*_1,47_ = 4.09, *P = *0.049). *P<0.05; ***P<0.001; ****P<0.0001 versus +/+ mice.

To evaluate the cognitive abilities of *Myk*/+ mice, we used two behavioural tests of associative learning and long-term (24-hour) memory. Fear conditioning tests an animal's ability to learn and remember an association between an environment and foot shock (contextual fear conditioning), or between an auditory tone cue (the conditioned stimulus, CS) and foot shock (cued fear conditioning). The procedure takes advantage of the natural tendency of rodents to suppress any movement besides respiration and heartbeat (freezing) in response to fearful stimuli. In the fear conditioning chamber, both genotypes showed similarly low levels of baseline freezing. In the contextual fear conditioning test, a day after mice had received a 1-mA footshock, both genotypes showed significantly increased levels of freezing relative to baseline freezing (*P<*0.0001), but *Myk*/+ mice showed a significantly lower level of freezing in the context than +/+ mice (Figure 3A). In the cued fear conditioning test, the freezing level of both genotypes in the altered context prior to presentation of the auditory tone (Pre-CS) was significantly greater than baseline freezing (*P<*0.0001), suggesting that both genotypes showed some fear response to the altered context. During presentation of the auditory tone (CS), both genotypes increased their freezing levels relative to Pre-CS freezing (*P<*0.0001), although *Myk*/+ mice showed a significantly lower level of CS freezing than +/+ mice (Figure 3A). To investigate the effect of a less noxious stimulus on the apparent fear conditioning deficit of *Myk*/+ mice, we subjected a fresh cohort of mice to fear conditioning with a 0.75 mA footshock. The freezing levels of both genotypes were reduced, but the significantly lower levels of contextual freezing and CS freezing of *Myk*/+ mice were maintained (Figure S3E). Despite the stressful nature of the fear conditioning test, *Myk*/+ mice did not exhibit convulsions during this procedure.

Acoustic startle is a motor reflex response to an intense loud noise stimulus. Given the reduced cued fear conditioning of *Myk*/+ mice, we assessed their acoustic startle response as a measure of gross hearing ability. There was no effect of genotype on startle responses to brief (40 ms) white noise bursts of 85–120 decibels (dB), but male *Myk*/+ mice showed an enhanced startle response at lower sound volumes (70–80 dB) (Figure S3F).

As a secondary test of learning and memory, we used a measure of associative learning known as conditioned taste aversion (CTA), which involves animals recognizing a novel taste as aversive when it has been associated with post-ingestive malaise. A novel taste (conditioned stimulus, CS; saccharin solution) was paired with the gastric malaise-inducing agent lithium chloride (unconditioned stimulus, US; LiCl). 24 hours following pairing with LiCl, the degree of CTA learning was determined by a CS consumption score (intake of saccharin/total fluid), a low score indicating relatively little consumption of the CS and, consequently, a strong CTA. After treatment with LiCl, both genotypes showed an aversion to saccharin compared to vehicle-treated mice, but *Myk*/+ mice had a higher CS consumption score and thus a weaker CTA than +/+ mice (Figure 3B). *Myk*/+ and +/+ mice consumed similar total levels of fluid in the test session (data not shown).

### Altered regional cerebral metabolism and functional connectivity in *Myk*/*+* mice

**Figure 4:**
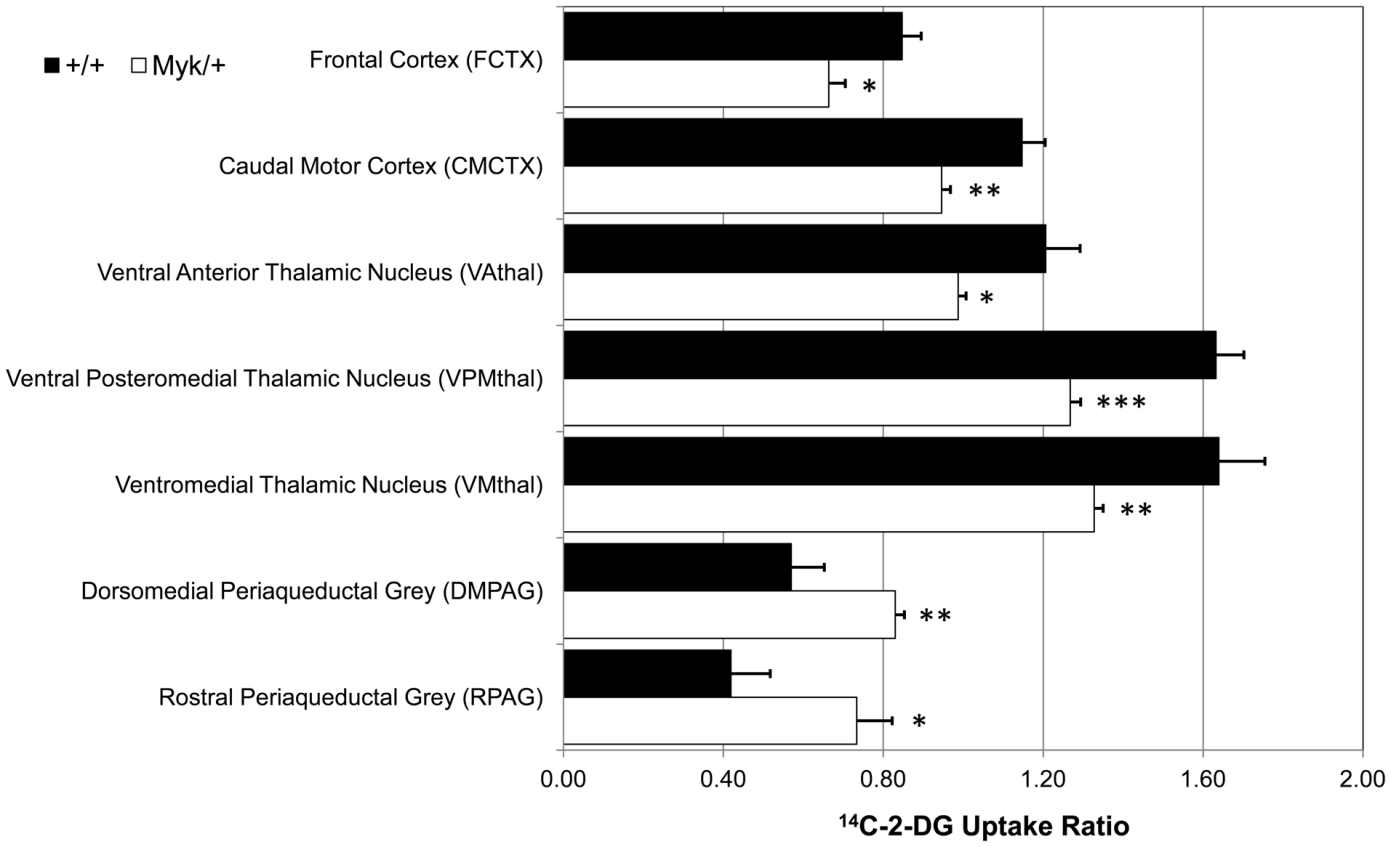
Significant alterations in overt local cerebral glucose utilization in *Myk*/+ mice. Data shown as mean ± SEM of the ^14^C-2-DG uptake ratio. *denotes *P<*0.05, **denotes *P<*0.01 and ***denotes *P<*0.001 significant genotype effect (Welch's *t*-test).

We undertook 2-DG imaging of the brains of *Myk*/+ mice to determine the effect of the I810N Na^+^,K^+^-ATPase α3 mutation on cerebral glucose metabolism. We found significantly decreased overt cerebral glucose metabolism in the frontal cortex (FCTX: −22%, *P = *0.016), multiple thalamic nuclei (ventral anterior nucleus [VAthal]: −18%, *P = *0.040; ventral posteromedial nucleus [VPMthal]: −22%, *P = *0.005; ventromedial nucleus [VMthal]: −19%, *P = *0.031) and caudal motor cortex (CMCTX: −18%, *P = *0.017) in *Myk*/+ as compared to +/+ mice. By contrast, cerebral glucose metabolism was profoundly increased in the periaqueductal grey (PAG) of *Myk*/+ as compared to +/+ mice (Rostral PAG [RPAG]: +75%, *P = *0.041 and dorsomedial PAG [DMPAG]: +46%, *P = *0.016) (Figure 4). Full data for overt cerebral glucose metabolism are shown in Table S1.

**Figure 5:**
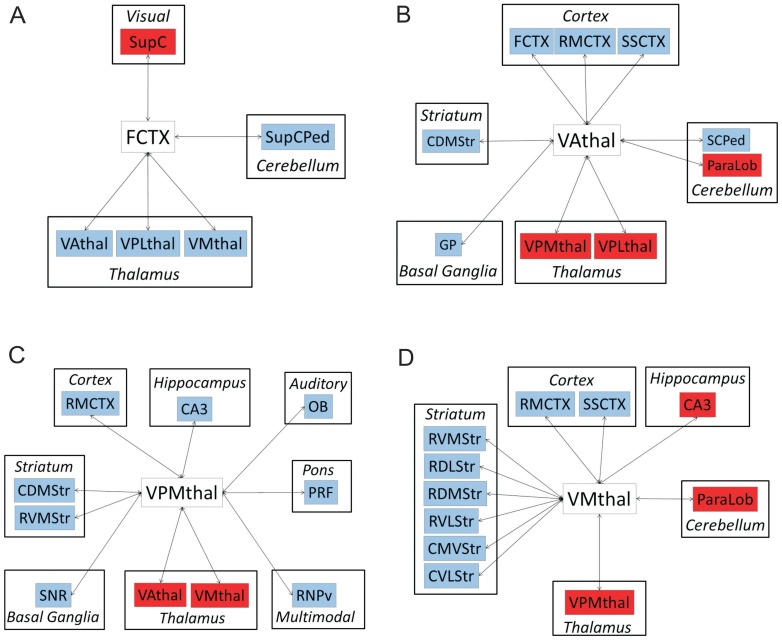
Thalamocortical, thalamostriatal and intrathalamic functional connectivity in *Myk*/+ mice. Summary diagrams showing altered functional connectivity of (A) frontal cortex (FCTX), (B) ventral anterior thalamic nucleus (VAthal), (C) ventromedial thalamic nucleus (VMthal), and (D) ventral posteromedial nucleus (VPMthal) in *Myk*/+ mice. Only regions where the 95% CI of the VIP exceeded 0.80, in either *Myk*/+ or +/+ mice, were considered to be functionally connected to the defined “seed” region of interest. The I810N *Myshkin* mutation-induced alterations in functional connectivity were analysed by permutation test (1000 random permutations of the real data) with significance set at *P<*0.05. Red denotes a significant increase, whereas blue denotes a significant decrease, in regional functional connectivity in *Myk*/+ mice relative to +/+.

Partial least squares regression (PLSR) analysis revealed decreased thalamocortical and thalamostriatal functional connectivity in *Myk*/+ mice relative to their +/+ littermates. Analysis of frontal cortex (FCTX) connectivity revealed a significant decrease in the functional connectivity of this region to multiple thalamic nuclei including the ventral anterior thalamic nucleus (VAthal), ventromedial thalamic nucleus (VMthal) and ventral posteromedial thalamic nucleus (VPMthal) in *Myk*/+ relative to +/+ mice. By contrast, the functional connectivity of the FCTX to the superior colliculus (SupC) was significantly enhanced in *Myk*/+ mice (Figure 5A).

Reduced thalamocortical functional connectivity in *Myk*/+ mice is further supported by the observation that all three thalamic nuclei considered as “seed” regions in PLSR analysis (VAthal, VPMthal and VMthal) showed reduced functional connectivity to multiple cortical regions, including the FCTX, rostromedial motor (RMCTX) and somatosensory cortex (SSCTX). In addition, all three thalamic regions showed significantly reduced connectivity to multiple subfields of the striatum, with connectivity to the caudal dorsomedial striatum (CDMStr) being significantly reduced for all three thalamic “seed” regions. By contrast, there was evidence for abnormally enhanced intrathalamic functional connectivity in *Myk*/+ mice that included enhanced connectivity between all three of the considered thalamic “seed” regions (Figure 5B–D). Full data for thalamocortical functional connectivity are shown in Table S2, S3, S4, S5.

**Figure 6:**
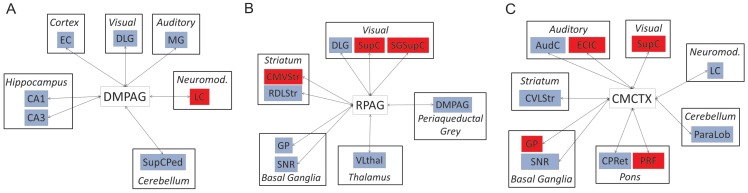
Altered periaqueductal grey and caudal motor cortex functional connectivity in *Myk*/+ mice. Summary diagrams showing altered functional connectivity of (A) dorsomedial (DMPAG) and (B) rostral (RPAG) periaqueductal grey, and (C) caudal motor cortex (CMCTX) in *Myk*/+ mice. Only regions where the 95% CI of the VIP exceeded 0.80, in either *Myk*/+ or +/+ mice, were considered to be functionally connected to the defined “seed” region of interest. The I810N *Myshkin* mutation-induced alterations in functional connectivity were analysed by permutation test (1000 random permutations of the real data) with significance set at *P<*0.05. Red denotes a significant increase, whereas blue denotes a significant decrease, in regional functional connectivity in *Myk*/+ mice relative to +/+.

The functional connectivity of the periaqueductal grey (PAG) was also profoundly altered in *Myk*/+ mice, as evidenced by the altered functional connectivity of the dorsomedial (DMPAG) and rostral (RPAG) periaqueductal grey (Figure 6). There was evidence that the functional connectivity between these two PAG regions was significantly decreased in *Myk*/+ mice. This resulted in the DMPAG showing increased functional connectivity to the locus coeruleus (LC), a primary source of noradrenergic innervation to the PAG, but a decreased functional connectivity to multiple hippocampal subfields (CA1, CA3) and the entorhinal cortex (EC), a cortical region with dense reciprocal connections to the hippocampus (Figure 6A). By contrast, the functional connectivity of the RPAG with the hippocampus was not altered. Rather, this PAG subfield, being functionally disconnected from the DMPAG in *Myk*/+ mice, showed reduced connectivity to regions of the basal ganglia (globus pallidus [GP], substantia nigra pars reticulata [SNR]) and the thalamus (VLthal). In addition, the RPAG showed increased connectivity to the superior colliculus (SupC and the superficial grey layer of the superior colliculus [SGSupC]) in *Myk*/+ mice relative to +/+ controls (Figure 6B).

The caudal motor cortex (CMCTX) showed a complex pattern of altered functional connectivity to multiple neural systems in *Myk*/+ mice. This included reduced functional connectivity to the LC, reduced connectivity to the striatum (caudal ventrolateral striatum [CVLStr]), and a contrasting enhancement in functional connectivity to components of the visual system (SupC) (Figure 6C). Full data for periaqueductal grey and caudal motor cortex functional connectivity are shown in Table S6, S7, S8.

### Elevated arterial blood pressure in *Myk*/*+* mice

Since chronic intracerebroventricular (ICV) infusion of the specific Na^+^,K^+^-ATPase inhibitor ouabain, but not the same dose given subcutaneously, has been shown to activate the orthosympathetic nervous system and elevate blood pressure in wild-type mice [30], we measured the heart rate and arterial blood pressure of *Myk*/+ mice. We found that *Myk*/+ mice had elevated mean, systolic, and diastolic blood pressure compared to +/+ littermates (Figure S4), whereas heart rate was not significantly different between genotypes (*Myk*/+ 578.7±33.8 bpm versus +/+ 555.0±10.2 bpm; *P = *0.269).

## Discussion

Overall, a consistent structure-phenotype relationship emerged from our comparative molecular modelling of AHC and RDP mutations affecting the same positions in Na^+^,K^+^-ATPase α3. The AHC mutations, D801N, I810S, I274N and D923Y, as well as the I810N *Myshkin* mutation, all bring about structural changes that severely affect capacity for efficient K^+^ movement along the narrow corridor of the K^+^ pore. The RDP mutations at the same positions, namely D801Y, I274T and D923N, have somewhat milder structural impacts that are likely to result in impaired K^+^ conductance, but not to the extent of the AHC mutations. This finding supports the notion that specific amino acid changes explain the relatively mild and the late onset clinical phenotype of RDP compared with AHC [6].

Examination of structural homologues of the mouse Na^+^,K^+^-ATPase α3 reveals that the cytoplasmic Na^+^ binding site of the rabbit sarcoplasmic calcium ATPase (PDB: 2DQS, Toyoshima and colleagues) is in an almost identical position, indeed just alongside when superimposed, to the cytoplasmic K^+^ binding site in the main modelling template used here, the spiny dogfish Na^+^,K^+^-ATPase (PDB: 2ZXE, [15]). There is no atomic information for the transmembrane region binding sites of Na^+^ for any of the Na^+^,K^+^-ATPases, though sections of the TM5, TM6 and TM8 regions have been implicated in cytoplasmically facing Na^+^ binding sites [31]–[33], which complements our deductions regarding the importance of E776 (TM5), D801 and I810 (TM6), and D923 (TM8) in K^+^ binding. It is conceivable that, when the Na^+^ binding sites are eventually elucidated, they may share structural elements with the K^+^ pore, the two cations perhaps using similar corridors on their way through the membrane. Our deductions from structural modelling regarding the impact of these mutations on K^+^ transport are balanced by awareness that the mutations may well also impact, or even primarily impact, upon Na^+^ transport. The disease phenotypes may be a manifestation of disruption of the movement of either or both cations.

Na^+^,K^+^-ATPases are functionally regulated by cyclic AMP-activated protein kinase A (PKA) phosphorylation on a well-conserved serine residue (S933 in α3; S943 in α1) in the cytoplasmic loop between TM8 and TM9 [34], [35]. S933 is the only well-characterized phosphorylation target in the Na^+^,K^+^-ATPase [34], but several other phosphorylation sites have been detected by mass spectrometry [36]. Although the change to a serine imparted by mutation I810S in AHC [7] raises the theoretical potential for novel phosphorylation to occur on the S810 mutant α3, the NetPhos online tool for predicting potential phosphorylation sites (www.cbs.dtu.dk/services/NetPhos) [37] does not predict an additional serine phosphorylation site on S810 (AHC) compared to N810 (*Myshkin*) and I810 (wild-type) Na^+^,K^+^-ATPase α3.

No animal model is currently available for *ATP1A3*-associated AHC. However, our finding that the I810N *Myshkin* mutation imparts similarly severe structural consequences upon pore architecture as the AHC mutation, I810S, establishes the aetiological relevance of *Myk*/+ mice to AHC. Based on this finding, we proceeded to analyse *Myk*/+ mice for signs of the permanent (non-episodic) symptoms of AHC.

Our observation that *Myk*/+ mice have a significantly shorter trunk and lower body weight than +/+ mice is consistent with the tendency of AHC patients to be of short stature and low weight, in comparison to mean values according to age [1], [4]. Decreased body size/weight has also been observed in other mice with ataxia and tremor, such as the classical neurological mutants *frissonnant* (*Kcnn2^fri^*) [38], *reeler* (*Reln^rl^*) [39], *trembler* (*Pmp22^Tr^*) [40] and *weaver* (*Kcnj6^wv^*) [41]. Studies to elucidate the cause of the low body weight found decreased circulating levels of IGF-I in *weaver* mice [41], but unaltered circulating levels of growth hormone (GH) in *reeler* mice [39]. It has been postulated that the restricted growth of AHC patients is almost certainly because of insufficient calorie intake caused by difficulties in feeding and swallowing [1], but a specific study of energy metabolism would be required to investigate the nutrition of *Myk*/+ mice.

In two large surveys of AHC patients, the most common permanent symptoms were ataxia and cognitive impairment [3], [4]. We observed that *Myk*/+ mice exhibit an unsteady, tremorous gait from about 4 weeks of age. We did not observe overt hemiplegia in *Myk*/+ mice, but their occasional splaying of the hindlimbs potentially models the episodes of bilateral weakness reported by 86.5% of AHC patients [4]. In common with AHC, episodic hemiplegia or hemiparesis is a clinical feature of familial hemiplegic migraine type 1 (FHM1), an autosomal dominant disorder caused by missense mutations in the *CACNA1A* gene encoding the pore-forming α_1A_-subunit of neuronal, voltage-gated Ca_V_2.1 calcium channels [42]. Homozygotes or heterozygotes of two knock-in FHM1 mouse models carrying human pathogenic missense mutations appear phenotypically normal and did not exhibit neurological deficits when assessed using a wire grip test and neurological examination protocols [43]. In common with *Myk*/+ mice, homozygous FHM1 mutant mice did not display overt hemiplegia, and only exhibited leaning and circling behaviour and wire grip deficits after topical application of KCl onto the occipital cortex following burr hole surgery [44].

We quantified the motor phenotype of *Myk*/+ mice in several behavioural tests that, in aggregate, assess overlapping aspects of sensorimotor function such as motor power, coordination, and postural stability. Gait analysis showed that the length and width of the forelimb stride and the length of the hindlimb stride are significantly smaller in female *Myk*/+ mice as compared to +/+ littermates, with no difference in the width of the hindlimb stride. However, adjustment of these data for the genotypic difference in body size revealed that, proportionately, *Myk*/+ mice walk with shorter strides and a broader base of the hindlimbs. For technical reasons, male *Myk*/+ mice were not available for the gait analysis, but the unsteady gait exhibited by *Myk*/+ mice of both sexes suggests that abnormal pawprint patterns may not be restricted to *Myk*/+ females.

*Myk*/+ mice performed poorly in the balance beam task that assesses a mouse's ability to maintain balance while traversing a narrow beam to reach a safe platform. In the accelerating rotarod test, mice are placed on the rod and their latency to fall provides a measurement of their motor coordination. *Myk*/+ mice had a significantly shorter latency to fall than +/+ mice on the first day of the rotarod test, demonstrating impaired motor coordination. However, *Myk*/+ mice showed no differences in performance on the four subsequent days of testing, demonstrating an intact ability to acquire new motor skills (motor learning). Mouse weight is reported to be a common confound of the rotarod test, heavy mice performing worse than light mice, such that genetic or lesion-induced weight loss can offset motor disability and potentially skew results [45]. Although the performance of *Myk*/+ mice on the rotarod may have been worse were it not for their significantly lower body weight, transgenic mouse models of DYT1 torsion dystonia and DYT11 myoclonic dystonia have also been reported to show deficits on the balance beam but normal motor learning on the rotarod [46]–[48].

*Myk*/+ mice showed deficits in contextual fear conditioning and cued fear conditioning in tests using two different footshock currents, with the higher amplitude footshock eliciting a stronger freezing response. The robust startle responses of *Myk*/+ mice to auditory stimuli of 70–120 dB in the acoustic startle test suggest that the reduced freezing of *Myk*/+ mice cannot be attributed to a reduced capacity to hear the 95-dB auditory tone during the fear conditioning procedure. Moreover, our previous finding that *Myk*/+ mice show normal head tracking in an optokinetic drum suggests that their vision is not impaired [13]. The mean freezing level of 55% of *Myk*/*+* mice in the cued fear conditioning test suggests that their ability to exhibit freezing was not grossly affected by their whole body tremor. Nevertheless, we measured conditioned taste aversion (CTA) as a secondary test of learning and memory that does not rely on an ability to suppress movement. We found that, compared to +/+ mice, *Myk*/+ mutants demonstrated a weaker CTA, a form of implicit memory that can be acquired even after massive damage to the hippocampus [49], [50].

By contrast with *Myk*/+ mutants, mice heterozygous for a point mutation in *Atp1a3* intron 4 (*Atp1a3* ^tm1Ling/+^) – which reduces hippocampal α3 expression by ∼60% and total brain Na^+^,K^+^-ATPase activity (α1 + α2 + α3) by ∼16% [12], [13] – do not exhibit restricted growth and visible neurological defects [51]. *Atp1a3* ^tm1Ling/+^ females exhibited deficits in the balance beam and accelerating rotarod tests only after exposure to restraint stress for five days [52]. The greater loss of Na^+^,K^+^-ATPase activity in *Myk*/+ mice offers a plausible, albeit speculative, explanation for their more severe neurological phenotype. Similar to *Myk*/+ mutants, *Atp1a3* ^tm1Ling/+^ mice showed increased locomotor activity in an open field and some impairment in learning and memory [13], [51]. While novel object recognition memory was normal in *Atp1a3* ^tm1Ling/+^ mice, they showed a learning and memory deficit in the Morris water maze [51], a spatial navigation task that requires mice to swim in a pool of opaque water. In the present study, we did test *Myk*/+ and +/+ mice in the Morris water maze, but have not presented the data here because there were genotypic differences in swimming behaviour; *Myk*/+ mice often displayed undirected swimming in tight circles (Video S3).

Another mouse model employs a pharmacological approach to perturb Na^+^,K^+^-ATPase function. Perfusion of ouabain into the cerebellum and basal ganglia has been found to induce mild dyskinesia in C57BL/6 mice, but when these mice were exposed to random electric footshocks in a warm environment (38°C) for 2 hours, 70% of them developed persistent dystonia and rigidity [53]. This pharmacological approach is limited by the ability to simultaneously target only two brain regions, and the similar affinities of the α2 and α3 isoforms for ouabain [54], thus precluding isoform specificity. Hence, *Myshkin* is the first mouse model with specific perturbation of Na^+^,K^+^-ATPase α3 to exhibit motor deficits in the absence of experimental stressors, thus demonstrating a greater similarity of the *Myk*/+ phenotype to AHC than RDP.

**Figure 7:**
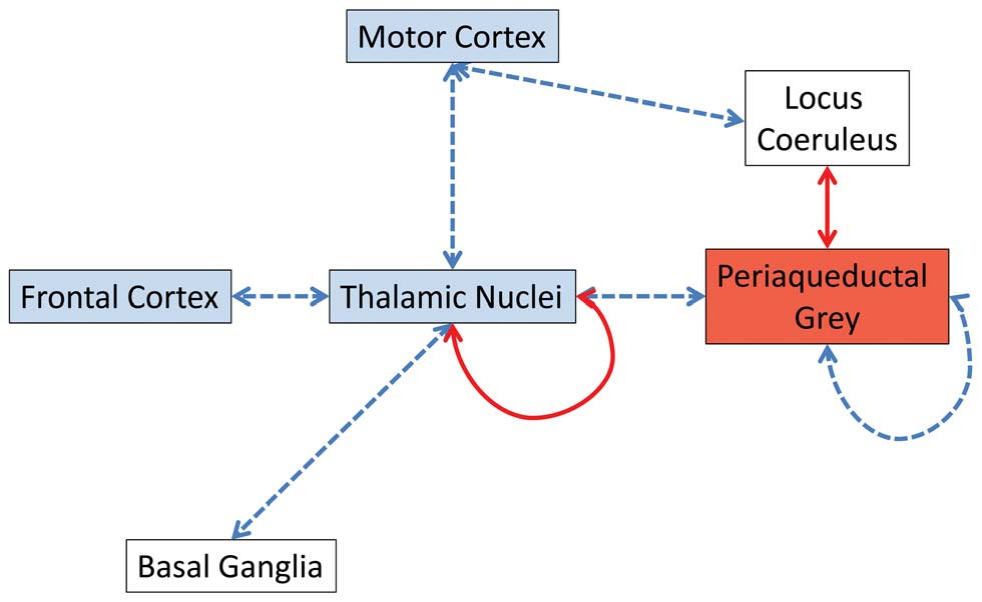
Summary diagram of alterations in brain system functional connectivity and overt alterations in regional cerebral glucose metabolism seen in *Myk*/+ mice. Blue shading of neural systems indicates a significant decrease in overt cerebral metabolism while red denotes a significant increase in overt cerebral metabolism (Figure 4). Blue/broken arrows indicate a decrease in functional connectivity between and within (periaqueductal grey subfields) neural systems in *Myk*/+ mice relative to +/+ littermates. Red/solid arrows indicate increased functional connectivity between and within (thalamic nuclei) neural systems in *Myk*/+ mice relative to +/+ littermates.

The most common finding of 2-DG PET scans of AHC patients is an interictal decrease in brain glucose metabolism [3], [5]. Our finding that glucose metabolism in the *Myshkin* brain was decreased in the frontal cortex, multiple thalamic nuclei and caudal motor cortex, and profoundly increased in the periaqueductal grey, is consistent with the variety of phenotypic abnormalities exhibited by *Myk*/+ mice, including the previously described neuronal hyperexcitability with increased susceptibility to epileptic seizures [12] and mania-related behaviours such as sleep disturbances and lengthened circadian period [13]. An immunohistochemical study of Na^+^,K^+^-ATPase α3 expression in the adult mouse brain showed that Na^+^,K^+^-ATPase α3 has a restricted distribution throughout the brain, with expression mainly in GABAergic neurones [55]. Among the brain structures with altered glucose metabolism for which Na^+^,K^+^-ATPase α3 expression data were available, Na^+^,K^+^-ATPase α3-positive cells were observed in the frontal association cortex, primary motor cortex, ventral anterior thalamic nucleus, ventral posteromedial thalamic nucleus, and dorsomedial periaqueductal grey, but not in the ventromedial thalamic nucleus [55]. Other brain structures, such as the basal ganglia and cerebellum involved in movement control, showed high expression of Na^+^,K^+^-ATPase α3 [55], but did not show altered glucose metabolism in *Myk*/+ mice. Therefore, the altered glucose metabolism was not confined to brain structures expressing Na^+^,K^+^-ATPase α3. Of the several brain regions previously found by MRI to have altered volumes in *Myk*/+ mice [12], only the thalamus was found to have altered glucose metabolism. Indeed, our data highlight the thalamus as being central to the compromised thalamocortical functioning in *Myk*/+ mice that includes a deficit in frontal cortex functioning (hypofrontality), directly mirroring that reported in AHC [3], [5], along with reduced thalamocortical functional connectivity (Figure 7). The elucidation of alterations in functional connectivity between defined brain regions and neural subsystems from 2-DG brain imaging data has previously been described [25], [56], [57].

The hypofrontality and frontal cortex dysconnectivity seen in *Myk*/+ mutants may directly contribute to the cognitive deficits we have seen in these mice, as these neural systems have an established role in associative learning [58], fear conditioning [59], [60] and other high level cognitive processes [56], [61]. The disrupted functional connectivity between the thalamus and basal ganglia and thalamus and motor cortex that we have identified in *Myk*/+ mutants is also consistent with aspects of the motor dysfunction we have observed in these mice. It is particularly interesting that *Myk*/+ mice show increased tremor, as evidence suggests that the regulation of thalamic circuitry by components of the basal ganglia plays a central role in regulating tremor amplitude [62], [63]. Overall, these data suggest that disrupted functional connectivity between these neural systems may underlie the motor symptoms of *Myk*/+ mice and, by extension, AHC patients.

AHC patients exhibit autonomic symptoms, such as alterations in skin colour, temperature, and sweating, concurrent with hemiplegia or in isolation [4]. The elevated mean, systolic, and diastolic blood pressure, but normal heart rate, of *Myk*/+ mice compared to +/+ littermates is similar to the effect on cardiovascular haemodynamics of chronic ICV infusion of ouabain in wild-type mice [30]. As the ubiquitously expressed α1 isoform is naturally resistant to ouabain in rodents [64], it is highly unlikely that it plays a role in ouabain-induced hypertension. In contrast, the α2 and α3 isoforms have a high affinity for ouabain and thereby could mediate the pressor effects of ouabain [54]. Studies utilizing *Atp1a2* ^tm3Ling^ mice homozygous for a ouabain-resistant α2 isoform, such that the only ouabain-sensitive isoform they express in the CNS is α3, found that ICV ouabain did not raise blood pressure in these mice [30]; this suggested that the pressor response to ICV ouabain is mediated solely by the α2 isoform in the brain. Nevertheless, our finding of hypertension in *Myk*/+ mice suggests that the α3 isoform may also play a role in the regulation of blood pressure. We previously found that the antihypertensive agent rostafuroxin [65], which antagonizes the inhibitory action of ouabain on Na^+^,K^+^-ATPase, reduced risk-taking behaviours in *Myk*/+ mice [13]. Blood pressure measurements in AHC patients have not been reported.

In summary, we found that *Myk*/+ mice carrying a Na^+^,K^+^-ATPase α3 missense mutation comparable to mutations observed in AHC patients exhibit motor and cognitive deficits that model the most common permanent symptoms of AHC. The *Myshkin* mutant is unique in that it is the first model described with an AHC-relevant phenotype associated with a Na^+^,K^+^-ATPase α3 missense mutation. Our results provide biological validation for heterozygous missense mutations in *ATP1A3* as a cause of AHC in humans, and highlight *Myshkin* as an aetiologically-relevant model system for the exploration of disease mechanisms and novel treatments in AHC. In this vein, we used *Myk*/+ mice to identify functional alterations in discrete brain regions and neural circuits that may underlie the overlapping symptoms presented by *Myk*/+ mice and AHC patients.

## Supporting Information

Figure S1 **Pathogenic mutations in mouse and human Na^+^,K^+^-ATPase α3.** (A) Alignment of the predicted Na^+^,K^+^-ATPase α3 protein sequences of *Homo sapiens* (OTTHUMP00000076119) and *Mus musculus* (ENSMUSP00000079691) surrounding residue I810 mutated in *Myshkin* mice. Residues mutated in RDP patients (red text), AHC patients (blue text) or *Myshkin* mice (green text) are shown, a grey background indicating residues mutated in both RDP and AHC, or in both AHC and *Myshkin*. Numbers flanking the alignment show the amino acid position. (B) Structural modelling of mouse Na^+^,K^+^-ATPase α3 wild-type, showing the positions of the mutated residues and predicted K^+^ ion binding sites. All the mutations modelled affect transmembrane helices bordering the K^+^ pore.
(PDF)

Figure S2 **Gait analysis.** (A) Relationship of trunk length to body weight in *Myk*/+ and +/+ female mice. Trendlines and Pearson correlation coefficients (*r*) are shown. (B) Unadjusted mean fore stride and hind stride distance (± SEM) of *Myk*/+ (*n* = 14) and +/+ (*n* = 17) female mice. There were significant main effects of genotype on fore stride width (*F*_1,30_ = 20.76, *P* = 0.0001), fore stride length (*F*_1,30_ = 42.65, *P* = 0.0001), and hind stride length (*F*_1,30_ = 42.45, *P* = 0.0001). ****P<0.0001 versus +/+ mice. (C) Relationship of fore stride width to trunk length; (D) fore stride length to trunk length; (E) hind stride width to trunk length; and (F) hind stride length to trunk length in *Myk*/+ and +/+ female mice. Trendlines and Pearson correlation coefficients (*r*) are shown.
(PDF)

Figure S3 **Behavioural analysis of** ***Myk*** **/+ mice.** (A) Motor learning on the accelerating rotarod. Mean latency (± SEM) of *Myk*/+ (*n* = 18) and +/+ (*n* = 21) mice to fall from a rotating rod at each block of three training trials on five consecutive days. There were significant main effects of sex (*F*_1,194_ = 41.05, *P* = 0.0001) and trial block (*F*_4,194_ = 13.09, *P* = 0.0001), but not genotype (*F*_1,194_ = 1.01, *P* = 0.316) or genotype x sex interaction (*F*_1,194_ = 0.03, *P* = 0.869). All genotype/sex groups demonstrated motor learning by having a longer latency to fall on Day 5 than on Day 1 of training (*P* = 0.0001). (B) Grip strength. Mean all-four limb and forelimb grip strength (± SEM) of male *Myk*/+ (*n* = 21), female *Myk*/+ (*n* = 9), male +/+ (*n* = 29) and female +/+ (*n* = 16) mice. Each mouse was given three trials. There was a significant main effect of genotype on the grip strength of all-four limbs in males (*F*_1,49_ = 10.15, *P* = 0.003). **P<0.01 versus +/+ males. (C) Relationship of all-four limb grip strength to body weight, and (D) forelimb grip strength to body weight in *Myk*/+ and +/+ mice. Trendlines and Pearson correlation coefficients (*r*) are shown. (E) Fear conditioning with 0.75-mA footshock. Mean freezing levels (± SEM) of *Myk*/+ (*n* = 22) and +/+ (*n* = 21) mice at baseline, during training, and in the contextual and cued conditioning tests. There were significant main effects of genotype on freezing in the context test (*F*_1,42_ = 16.27, *P = *0.0001), and in the cue test before (Pre-CS; *F*_1,42_ = 6.62, *P = *0.014) and during (CS; *F*_1,42_ = 14.23, *P = *0.001) presentation of the auditory tone. *P<0.05; ***P<0.001; ****P<0.0001 versus +/+ mice. (F) Acoustic startle response. Mean amplitude of startle response (± SEM) of *Myk*/+ males (*n = *21), *Myk*/+ females (*n = *17), +/+ males (*n = *29) and +/+ females (*n = *26) to auditory stimuli at volumes ranging from 70 to 120 dB. There were significant main effects of genotype (*F*_1,1022_ = 5.25, *P = *0.022), sex (*F*_1,1022_ = 35.34, *P = *0.0001), volume (*F*_10,1022_ = 20.12, *P = *0.0001), genotype x sex interaction (*F*_1,1022_ = 7.65, *P = *0.006) and genotype x volume interaction (*F*_10,1022_ = 3.39, *P = *0.0001). ****P<0.0001 versus +/+ males.
(PDF)

Figure S4 **Elevated arterial blood pressure in** ***Myk*** **/+ mice.** Mean, systolic, and diastolic arterial pressure (± SEM) in millimetres of mercury (mm Hg) of *Myk*/+ (*n = *3) and +/+ (*n = *3) male mice. There were significant main effects of genotype on mean arterial pressure (Mean BP; *P = *0.015), systolic arterial pressure (Systolic BP; *P = *0.004) and diastolic arterial pressure (Diastolic BP; *P = *0.034). *P<0.05; ***P<0.001 versus +/+ mice.
(PDF)

Table S1 **Full data for overt cerebral glucose metabolism.** Bold denotes regions showing significantly different overt cerebral metabolism in *Myk*/+ as compared to +/+ mice. *denotes *P*<0.05, **denotes *P*<0.01 and ***denotes *P*<0.001 significant difference from wild-type control (Welch's *t*-test).
(PDF)

Table S2 **Full data for frontal cortex (FCTX) functional connectivity.** Data shown as the mean ± SEM of the VIP statistic as determined through PLSR analysis. Bold denotes significant functional connection with the defined seed region in given experimental group (95% CI VIP>0.80). *denotes *P*<0.05 significant difference in VIP statistic between genotypes (1000 random permutations). Red highlights regions showing increased and blue decreased functional connectivity with the given seed brain region in *Myshkin* (*Myk*/+) mice relative to wild-type (+/+) controls.
(PDF)

Table S3 **Full data for ventral anterior thalamus (VAthal) functional connectivity.** Data shown as the mean ± SEM of the VIP statistic as determined through PLSR analysis. Bold denotes significant functional connection with the defined seed region in given experimental group (95% CI VIP>0.80). *denotes *P*<0.05 significant difference in VIP statistic between genotypes (1000 random permutations). Red highlights regions showing increased and blue decreased functional connectivity with the given seed brain region in *Myshkin* (*Myk*/+) mice relative to wild-type (+/+) controls.
(PDF)

Table S4 **Full data for ventromedial thalamus (VMthal) functional connectivity.** Data shown as the mean ± SEM of the VIP statistic as determined through PLSR analysis. Bold denotes significant functional connection with the defined seed region in given experimental group (95% CI VIP>0.80). *denotes *P*<0.05 significant difference in VIP statistic between genotypes (1000 random permutations). Red highlights regions showing increased and blue decreased functional connectivity with the given seed brain region in *Myshkin* (*Myk*/+) mice relative to wild-type (+/+) controls.
(PDF)

Table S5 **Full data for ventral posteromedial nucleus (VPMthal) functional connectivity.** Data shown as the mean ± SEM of the VIP statistic as determined through PLSR analysis. Bold denotes significant functional connection with the defined seed region in given experimental group (95% CI VIP>0.80). *denotes *P*<0.05 significant difference in VIP statistic between genotypes (1000 random permutations). Red highlights regions showing increased and blue decreased functional connectivity with the given seed brain region in *Myshkin* (*Myk*/+) mice relative to wild-type (+/+) controls.
(PDF)

Table S6 **Full data for dorsomedial periaqueductal grey (DMPAG) functional connectivity.** Data shown as the mean ± SEM of the VIP statistic as determined through PLSR analysis. Bold denotes significant functional connection with the defined seed region in given experimental group (95% CI VIP>0.80). *denotes *P*<0.05 significant difference in VIP statistic between genotypes (1000 random permutations). Red highlights regions showing increased and blue decreased functional connectivity with the given seed brain region in *Myshkin* (*Myk*/+) mice relative to wild-type (+/+) controls.
(PDF)

Table S7 **Full data for rostral periaqueductal grey (RPAG) functional connectivity.** Data shown as the mean ± SEM of the VIP statistic as determined through PLSR analysis. Bold denotes significant functional connection with the defined seed region in given experimental group (95% CI VIP>0.80). *denotes *P*<0.05 significant difference in VIP statistic between genotypes (1000 random permutations). Red highlights regions showing increased and blue decreased functional connectivity with the given seed brain region in *Myshkin* (*Myk*/+) mice relative to wild-type (+/+) controls.
(PDF)

Table S8 **Full data for caudal motor cortex (CMCTX) functional connectivity.** Data shown as the mean ± SEM of the VIP statistic as determined through PLSR analysis. Bold denotes significant functional connection with the defined seed region in given experimental group (95% CI VIP>0.80). *denotes *P*<0.05 significant difference in VIP statistic between genotypes (1000 random permutations). Red highlights regions showing increased and blue decreased functional connectivity with the given seed brain region in *Myshkin* (*Myk*/+) mice relative to wild-type (+/+) controls.
(PDF)

Video S1 **Movement of representative** ***Myk*** **/+ and +/+ mice in the home cage.** Note the unsteady, tremorous gait of the *Myk*/+ mouse. [AVI file; Duration: 00∶00∶30].
(AVI)

Video S2 **Representative** ***Myk*** **/+ mouse with splayed hindlimbs when walking.** [AVI file; Duration: 00∶00∶10].
(AVI)

Video S3 **Abnormal swimming behaviour of representative** ***Myk*** **/+ mouse.** [AVI file; Duration: 00∶00∶33].
(AVI)
