# Supplementary material for: Alternating Hemiplegia of Childhood-Related Neural and Behavioural Phenotypes in Na+,K+-ATPase α3 Missense Mutant Mice
Source: PLoS One. 2013 Mar 20;8(3):e60141. doi: 10.1371/journal.pone.0060141 (PMC3603922; doi:10.1371/journal.pone.0060141)

Supplementary Figure 1

A

Homo sapiens	751	GVEEGRLIFDNLKKSIAYTLTSNIPEITPFLLFIMANIPLPLGTITILCIDLGTDMVPAISLAYEAAESD	820
Homo sapiens	751	GVEEGRLIFDNLKKSIAYTLTSNIPEITPFLLFIMANIPLPLGTITILCIDLGTDMVPAISLAYEAAESD	820
Mus musculus	751	GVEEGRLIFDNLKKSIAYTLTSNIPEITPFLLFIMANIPLPLGTITILCIDLGTDMVPAISLAYEAAESD	820

G755C/S	AHC
R756H	RDP (infant)
I758S	RDP
S772R	AHC
N773I/S	AHC
F780L	RDP
D801N	AHC
D801Y	RDP
M806R	AHC
I810S	AHC
I810N	*Myshkin*
S811P	AHC
E815K	AHC

B

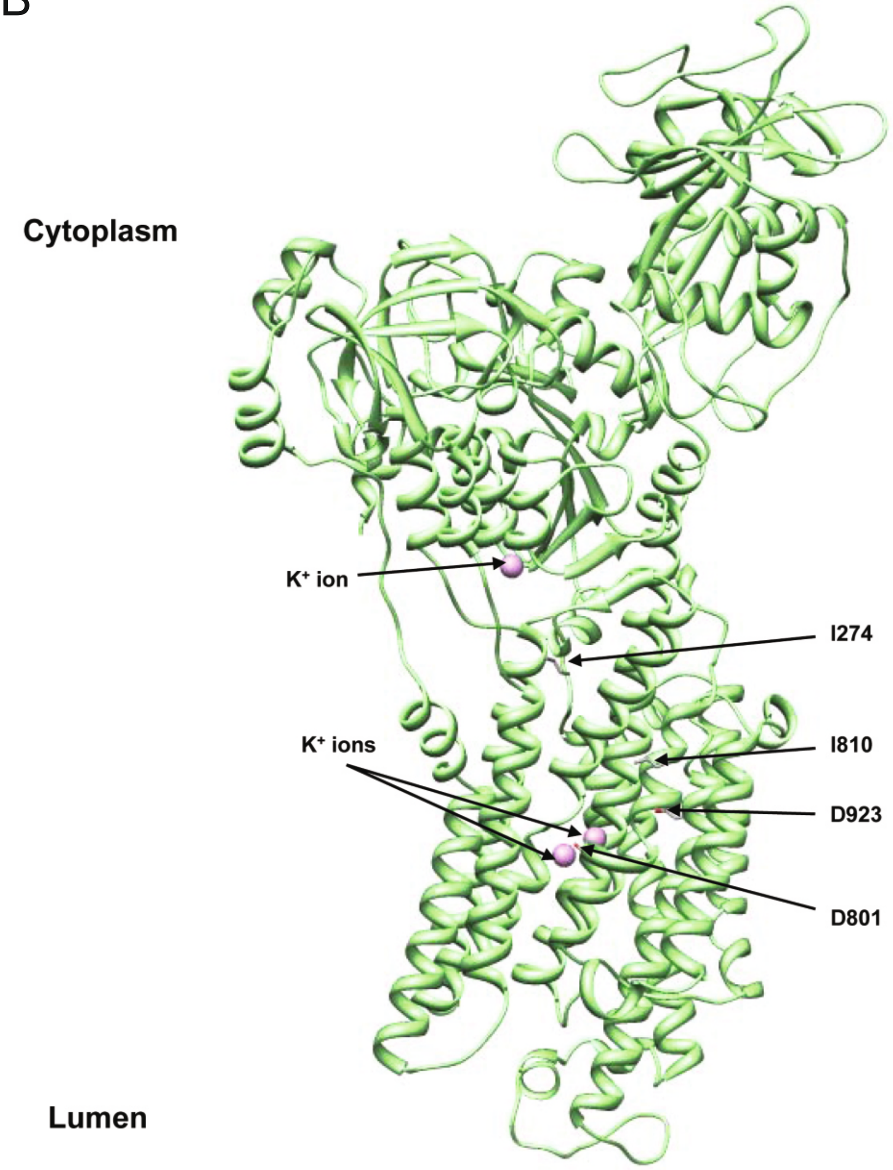

Supplement: Figure S1 — Pathogenic mutations in mouse and human Na+,K+-ATPase α3. (A) Alignment of the predicted Na+,K+-ATPase α3 protein sequences of Homo sapiens (OTTHUMP00000076119) and Mus musculus (ENSMUSP00000079691) surrounding residue I810 mutated in Myshkin mice. Residues mutated in RDP patients (red text), AHC patients (blue text) or Myshkin mice (green text) are shown, a grey background indicating residues mutated in both RDP and AHC, or in both AHC and Myshkin. Numbers flanking the alignment show the amino acid position. (B) Structural modelling of mouse Na+,K+-ATPase α3 wild-type, showing the positions of the mutated residues and predicted K+ ion binding sites. All the mutations modelled affect transmembrane helices bordering the K+ pore. (PDF) [file pone.0060141.s001.pdf]
