# Supplementary figures and images for: Alternating Hemiplegia of Childhood-Related Neural and Behavioural Phenotypes in Na+,K+-ATPase α3 Missense Mutant Mice
Source: PLoS One. 2013 Mar 20;8(3):e60141. doi: 10.1371/journal.pone.0060141 (PMC3603922; doi:10.1371/journal.pone.0060141)

# Supplementary Figure 2

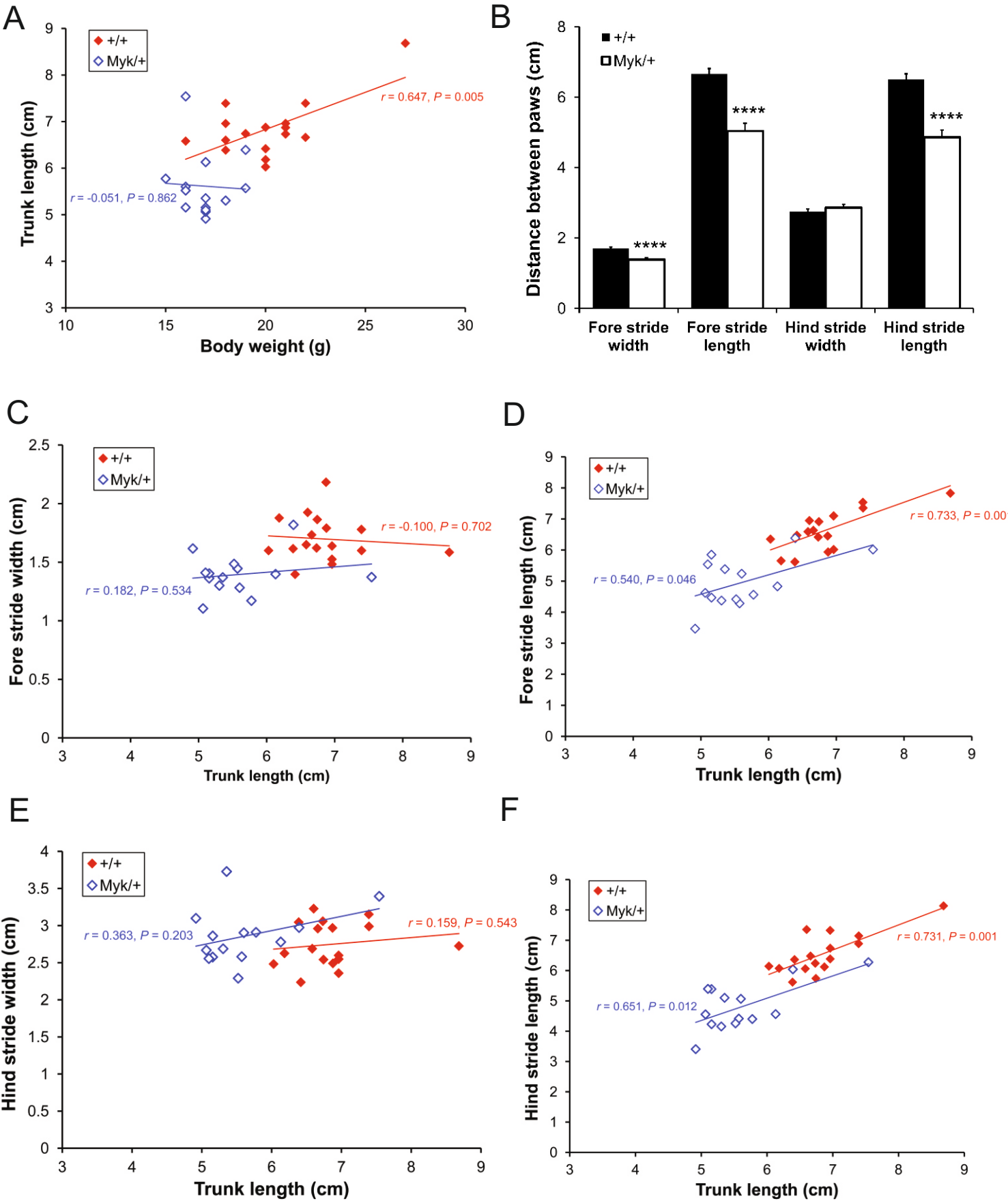

Supplement: Figure S2 — Gait analysis. (A) Relationship of trunk length to body weight in Myk/+ and +/+ female mice. Trendlines and Pearson correlation coefficients (r) are shown. (B) Unadjusted mean fore stride and hind stride distance (± SEM) of Myk/+ (n = 14) and +/+ (n = 17) female mice. There were significant main effects of genotype on fore stride width (F 1,30 = 20.76, P = 0.0001), fore stride length (F 1,30 = 42.65, P = 0.0001), and hind stride length (F 1,30 = 42.45, P = 0.0001). ****P<0.0001 versus +/+ mice. (C) Relationship of fore stride width to trunk length; (D) fore stride length to trunk length; (E) hind stride width to trunk length; and (F) hind stride length to trunk length in Myk/+ and +/+ female mice. Trendlines and Pearson correlation coefficients (r) are shown. (PDF) [file pone.0060141.s002.pdf]

# Supplementary Figure 3

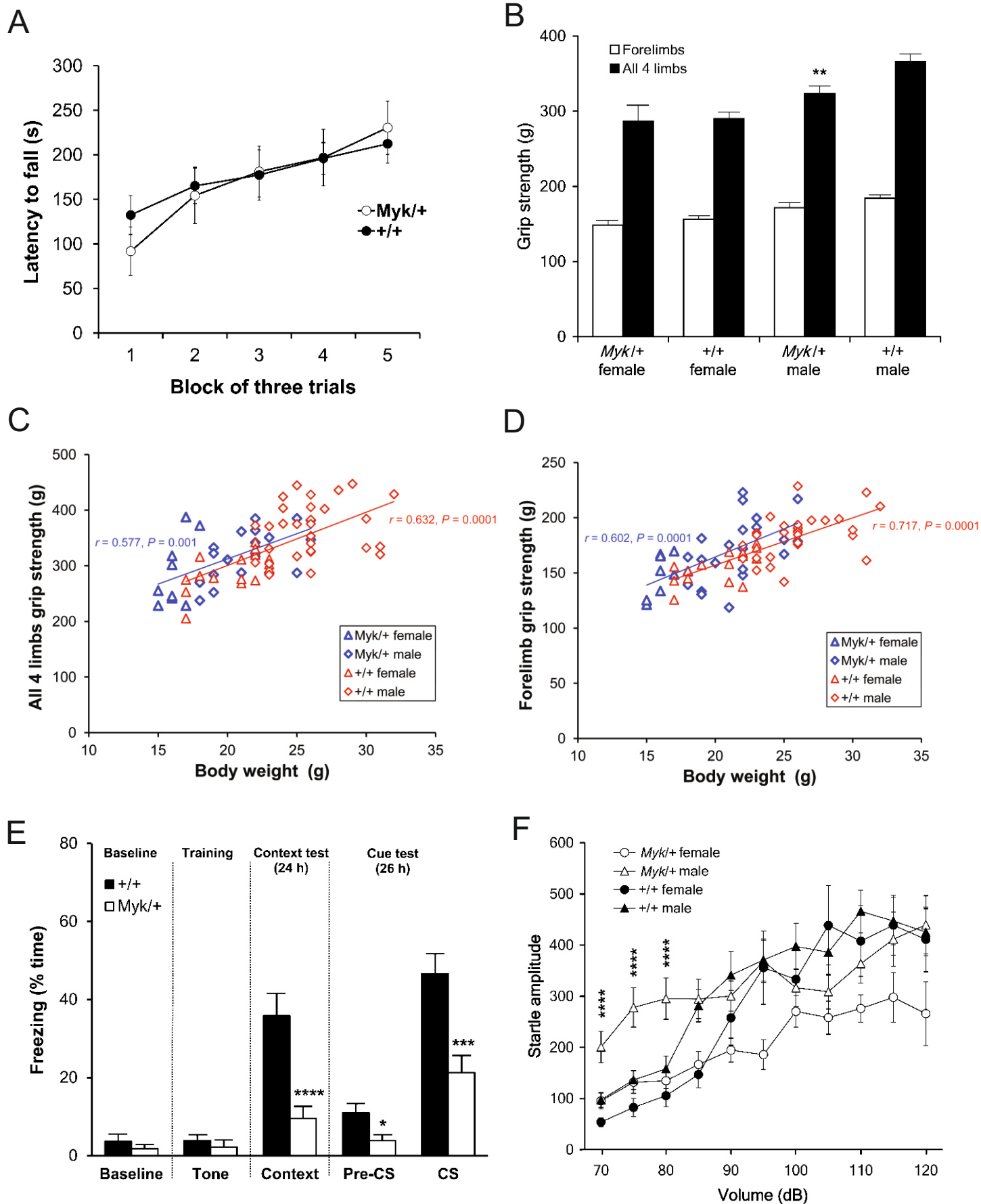

Supplement: Figure S3 — Behavioural analysis of Myk /+ mice. (A) Motor learning on the accelerating rotarod. Mean latency (± SEM) of Myk/+ (n = 18) and +/+ (n = 21) mice to fall from a rotating rod at each block of three training trials on five consecutive days. There were significant main effects of sex (F 1,194 = 41.05, P = 0.0001) and trial block (F 4,194 = 13.09, P = 0.0001), but not genotype (F 1,194 = 1.01, P = 0.316) or genotype x sex interaction (F 1,194 = 0.03, P = 0.869). All genotype/sex groups demonstrated motor learning by having a longer latency to fall on Day 5 than on Day 1 of training (P = 0.0001). (B) Grip strength. Mean all-four limb and forelimb grip strength (± SEM) of male Myk/+ (n = 21), female Myk/+ (n = 9), male +/+ (n = 29) and female +/+ (n = 16) mice. Each mouse was given three trials. There was a significant main effect of genotype on the grip strength of all-four limbs in males (F 1,49 = 10.15, P = 0.003). **P<0.01 versus +/+ males. (C) Relationship of all-four limb grip strength to body weight, and (D) forelimb grip strength to body weight in Myk/+ and +/+ mice. Trendlines and Pearson correlation coefficients (r) are shown. (E) Fear conditioning with 0.75-mA footshock. Mean freezing levels (± SEM) of Myk/+ (n = 22) and +/+ (n = 21) mice at baseline, during training, and in the contextual and cued conditioning tests. There were significant main effects of genotype on freezing in the context test (F 1,42 = 16.27, P = 0.0001), and in the cue test before (Pre-CS; F 1,42 = 6.62, P = 0.014) and during (CS; F 1,42 = 14.23, P = 0.001) presentation of the auditory tone. *P<0.05; ***P<0.001; ****P<0.0001 versus +/+ mice. (F) Acoustic startle response. Mean amplitude of startle response (± SEM) of Myk/+ males (n = 21), Myk/+ females (n = 17), +/+ males (n = 29) and +/+ females (n = 26) to auditory stimuli at volumes ranging from 70 to 120 dB. There were significant main effects of genotype (F 1,1022 = 5.25, P = 0.022), sex (F 1,1022 = 35.34, P = 0.0001), volume (F 10,10 [file pone.0060141.s003.pdf]

## Supplementary Figure 4

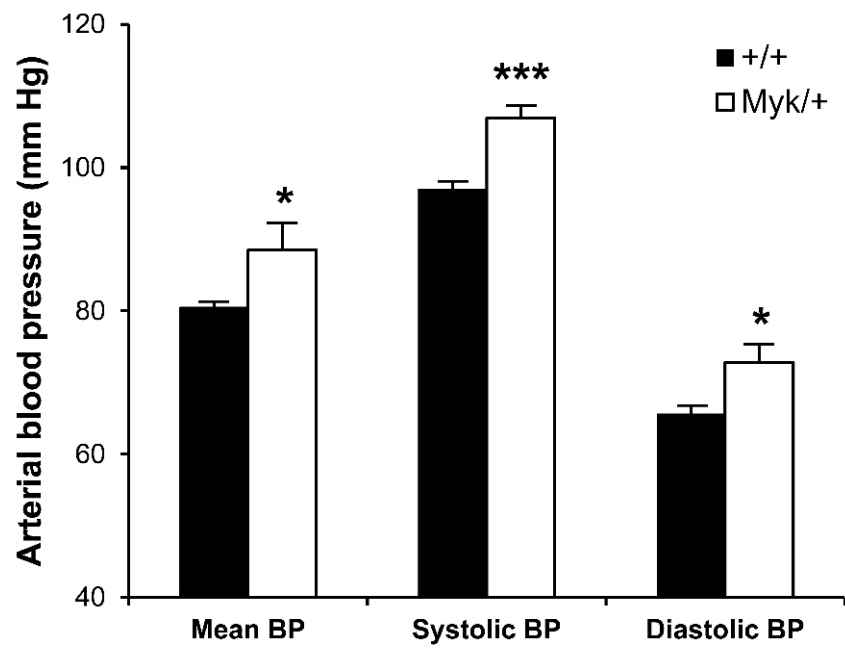

Supplement: Figure S4 — Elevated arterial blood pressure in Myk /+ mice. Mean, systolic, and diastolic arterial pressure (± SEM) in millimetres of mercury (mm Hg) of Myk/+ (n = 3) and +/+ (n = 3) male mice. There were significant main effects of genotype on mean arterial pressure (Mean BP; P = 0.015), systolic arterial pressure (Systolic BP; P = 0.004) and diastolic arterial pressure (Diastolic BP; P = 0.034). *P<0.05; ***P<0.001 versus +/+ mice. (PDF) [file pone.0060141.s004.pdf]
