# Supplementary material for: Alternating Hemiplegia of Childhood-Related Neural and Behavioural Phenotypes in Na+,K+-ATPase α3 Missense Mutant Mice
Source: PLoS One. 2013 Mar 20;8(3):e60141. doi: 10.1371/journal.pone.0060141 (PMC3603922; doi:10.1371/journal.pone.0060141)
Supplement: Table S1 — Full data for overt cerebral glucose metabolism. Bold denotes regions showing significantly different overt cerebral metabolism in Myk/+ as compared to +/+ mice. *denotes P<0.05, **denotes P<0.01 and ***denotes P<0.001 significant difference from wild-type control (Welch's t-test). (PDF) [file pone.0060141.s005.pdf]

Supplementary Table 1. Full data for overt cerebral metabolism.

Brain Region	Wild-type (+/+)			*Myshkin* (*Myk*/+)		
	mean		SEM	mean		SEM
Cortex						
**Frontal (FCTX)**	**0.85**	**±**	**0.05**	**0.66***	**±**	**0.04**
**Caudal Motor (CMCTX)**	**1.15**	**±**	**0.06**	**0.95****	**±**	**0.02**
Rostral Motor (RMCTX)	1.06	±	0.08	0.98	±	0.02
Somatosensory (SSCTX)	1.12	±	0.09	0.96	±	0.01
Entorhinal (EC)	0.71	±	0.08	0.59	±	0.02
Dorsolateral Entorhinal (DLECTX)	0.89	±	0.11	0.88	±	0.10
Thalamus						
**Ventral Anterior Nucleus (VAthal)**	**1.21**	**±**	**0.09**	**0.99***	**±**	**0.02**
Ventrolateral Nucleus (VLthal)	1.12	±	0.09	0.97	±	0.01
Ventral Posterolateral Nucleus (VPLthal)	1.34	±	0.08	1.18	±	0.03
**Ventral Posteromedial Nucleus (VPMthal)**	**1.63**	**±**	**0.07**	**1.27*****	**±**	**0.03**
**Ventromedial Nucleus (VMthal)**	**1.64**	**±**	**0.12**	**1.33****	**±**	**0.02**
Basal Ganglia						
Rostral Ventromedial Striatum (RVMStr)	1.01	±	0.08	1.01	±	0.02
Rostral Dorsolateral Striatum (RDLStr)	1.19	±	0.09	1.18	±	0.02
Rostral Dorsomedial Striatum (RDMStr)	1.00	±	0.08	1.08	±	0.04
Rostral Ventrolateral Striatum (RVLStr)	1.16	±	0.07	1.07	±	0.02
Caudal Centromedial Striatum (CCMStr)	1.22	±	0.08	1.09	±	0.03
Caudal Dorsolateral Striatum (CDLStr)	1.17	±	0.05	1.05	±	0.03
Caudal Dorsomedial Striatum (CDMStr)	1.06	±	0.07	0.94	±	0.03
Caudal Ventrolateral Striatum (CVLStr)	1.22	±	0.06	1.14	±	0.02
Globus Pallidus (GP)	0.89	±	0.05	0.79	±	0.03
Substantia Nigra pars Reticulata (SNR)	0.73	±	0.06	0.67	±	0.04
Hippocampus						
CA1	0.75	±	0.08	0.64	±	0.02
CA3	0.65	±	0.02	0.75	±	0.04
Auditory						
Auditory (AudC)	0.96	±	0.04	0.88	±	0.02
Dorsal Cortex of the Inferior Colliculus (DCIC)	1.44	±	0.27	0.93	±	0.07
External Cortex of the Inferior Colliculus (ECIC)	1.88	±	0.24	1.99	±	0.19
Medial Geniculate Nucleus (MG)	0.80	±	0.07	0.90	±	0.02
Olivary Body (OB)	0.89	±	0.08	0.88	±	0.07
Visual						
Dorsolateral Geniculate (DLG)	0.83	±	0.04	0.74	±	0.01
Superior Colliculus (SupC)	0.91	±	0.06	0.97	±	0.03
Superficial Grey Layer of the Superior Colliculus (SGSupC)	0.95	±	0.14	1.12	±	0.05
Neuromodulatory						
Median Raphe (MR)	1.43	±	0.14	1.57	±	0.07
Locus Coeruleus (LC)	0.78	±	0.08	0.82	±	0.03
Periaqueductal Grey						
Caudal PAG (CPAG)	0.57	±	0.11	0.80	±	0.04
**Dorsomedial PAG (DMPAG)**	**0.57**	**±**	**0.08**	**0.83****	**±**	**0.02**
**Rostral PAG (RPAG)**	**0.42**	**±**	**0.10**	**0.73***	**±**	**0.09**
Cerebellum						
Superior Cerebellar Peduncle (SupCPed)	0.96	±	0.09	0.75	±	0.03
Paramedian Lobule (PML)	0.95	±	0.04	0.96	±	0.17
Deep Cerebellar Nuclei (DCN)	0.98	±	0.06	0.98	±	0.10
Pons						

Gigantocellular Reticular Nucleus (GRt)	0.86	±	0.09	0.88	±	0.06
Caudal Pontine Reticular Nucleus (CPRt)	1.16	±	0.09	1.35	±	0.05
Pontine Reticular Formation (PRF)	0.82	±	0.06	0.88	±	0.05
Multimodal						
Red Nucleus Parvocellular (RNPv)	1.02	±	0.07	1.10	±	0.03
Red Nucleus Magnocellular (RNMn)	1.08	±	0.04	1.09	±	0.04
Medial Amygdala (MeA)	0.82	±	0.08	0.87	±	0.04
Medial Vestibular nucleus (MVN)	1.09	±	0.13	1.26	±	0.09

Bold denotes regions showing significantly different overt cerebral metabolism in *Myk*/+ as compared to +/+ mice. *denotes $P < 0.05$, **denotes $P < 0.01$ and ***denotes $P < 0.001$ significant difference from wild-type control (Welch's *t*-test).
