# Supplementary material for: Alternating Hemiplegia of Childhood-Related Neural and Behavioural Phenotypes in Na+,K+-ATPase α3 Missense Mutant Mice
Source: PLoS One. 2013 Mar 20;8(3):e60141. doi: 10.1371/journal.pone.0060141 (PMC3603922; doi:10.1371/journal.pone.0060141)
Supplement: Table S2 — Full data for frontal cortex (FCTX) functional connectivity. Data shown as the mean ± SEM of the VIP statistic as determined through PLSR analysis. Bold denotes significant functional connection with the defined seed region in given experimental group (95% CI VIP>0.80). *denotes P<0.05 significant difference in VIP statistic between genotypes (1000 random permutations). Red highlights regions showing increased and blue decreased functional connectivity with the given seed brain region in Myshkin (Myk/+) mice relative to wild-type (+/+) controls. (PDF) [file pone.0060141.s006.pdf]

Supplementary Table 2. Full data for frontal cortex (FCTX) functional connectivity.

Brain Region	Wild-type (+/+)			*Myshkin* (*Myk*/+)		
	mean		SEM	mean		SEM
Caudal Motor (CMCTX)	**0.88**	**±**	**0.10**	0.52	±	0.02
Rostral Motor (RMCTX)	**1.84**	**±**	**0.04**	**1.43**	**±**	**0.04**
Rostral Ventromedial Striatum (RVMStr)	**1.73**	**±**	**0.05**	**1.91**	**±**	**0.03**
Rostral Dorsolateral Striatum (RDLStr)	**1.53**	**±**	**0.05**	**1.85**	**±**	**0.02**
Rostral Dorsomedial Striatum (RDMStr)	**1.52**	**±**	**0.05**	**1.74**	**±**	**0.04**
Rostral Ventrolateral Striatum (RVLStr)	**1.64**	**±**	**0.05**	**1.68**	**±**	**0.06**
Caudal Centromedial Striatum (CCMStr)	0.67	±	0.09	0.49	±	0.03
Caudal Dorsolateral Striatum (CDLStr)	0.75	±	0.11	0.49	±	0.04
Caudal Dorsomedial Striatum (CDMStr)	0.59	±	0.07	0.75	±	0.03
Caudal Ventrolateral Striatum (CVLStr)	0.47	±	0.05	0.44	±	0.03
Globus Pallidus (GP)	0.65	±	0.05	**0.94**	**±**	**0.06**
Olivary Body (OB)	**1.25**	**±**	**0.05**	**0.93**	**±**	**0.05**
Auditory (AudC)	0.54	±	0.07	0.42*	±	0.05
Substantia Nigra pars Reticulata (SNR)	0.61	±	0.07	**0.85**	**±**	**0.07**
CA3	0.69	±	0.08	0.31*	±	0.15
Somatosensory (SSCTX)	0.77	±	0.07	0.30	±	0.12
Ventral Anterior Nucleus (VAthal)	**1.42**	**±**	**0.03**	0.41*	±	0.06
Ventrolateral Nucleus (VLthal)	**1.28**	**±**	**0.03**	0.75*	±	0.06
Dorsolateral Geniculate (DLG)	0.32	±	0.08	0.58	±	0.04
CA1	0.58	±	0.04	0.83	±	0.07
Entorhinal (EC)	0.62	±	0.08	**0.99**	**±**	**0.07**
Rostral PAG (RPAG)	**0.86**	**±**	**0.05**	0.77	±	0.11
Pontine Reticular Formation (PRF)	0.74	±	0.05	0.31	±	0.03
Dorsolateral Entorhinal (DLECTX)	**0.95**	**±**	**0.06**	**1.14**	**±**	**0.06**
External Cortex of the Inferior Colliculus (ECIC)	**0.99**	**±**	**0.10**	**1.09**	**±**	**0.06**
Median Raphe (MR)	0.50	±	0.09	0.41	±	0.03
Superior Colliculus (SupC)	**0.84**	**±**	**0.06**	**1.81***	**±**	**0.03**
Caudal Pontine Reticular Nucleus (CPRt)	**0.89**	**±**	**0.08**	0.67	±	0.04
Superficial Grey Layer of the Superior Colliculus (SGSupC)	**0.85**	**±**	**0.09**	**1.06**	**±**	**0.10**
Caudal PAG (CPAG)	**1.13**	**±**	**0.06**	0.61	±	0.05
Locus Coeruleus (LC)	0.61	±	0.05	**0.99**	**±**	**0.03**
Dorsal Cortex of the Inferior Colliculus (DCIC)	0.55	±	0.06	0.79	±	0.05
Gigantocellular Reticular Nucleus (GRt)	**1.15**	**±**	**0.05**	**1.38**	**±**	**0.06**
Medial Vestibular nucleus (MVN)	**0.84**	**±**	**0.05**	**1.67**	**±**	**0.05**
Superior Cerebellar Peduncle (SupCPed)	**1.37**	**±**	**0.06**	0.42*	±	0.04
Dorsomedial PAG (DMPAG)	0.73	±	0.06	**1.38**	**±**	**0.06**
Medial Geniculate Nucleus (MG)	0.43	±	0.03	0.58	±	0.05
Paramedian Lobule (PML)	**1.42**	**±**	**0.04**	**0.96**	**±**	**0.07**
Red Nucleus Parvocellular (RNPv)	0.62	±	0.03	0.44	±	0.07
Red Nucleus Magnocellular (RNMn)	0.48	±	0.04	0.35*	±	0.09
Deep Cerebellar Nuclei (DCN)	**0.93**	**±**	**0.04**	0.72	±	0.04
Medial Amygdala (MeA)	**1.63**	**±**	**0.05**	**1.41**	**±**	**0.06**
Ventral Posterolateral Nucleus (VPLthal)	**1.01**	**±**	**0.06**	0.42*	±	0.06
Ventral Posteromedial Nucleus (VPMthal)	0.52	±	0.07	0.42	±	0.04
Ventromedial Nucleus (VMthal)	**1.28**	**±**	**0.04**	**1.13**	**±**	**0.05**

Data shown as the mean ± SEM of the VIP statistic as determined through PLSR analysis. Bold denotes significant functional connection with the defined seed region in given experimental group (95% CI VIP>0.80). *denotes *P* < 0.05 significant difference in VIP statistic between genotypes (1000 random permutations). Red highlights regions showing increased and blue decreased functional connectivity with the given seed brain region in *Myshkin* (*Myk*/+) mice relative to wild-type (+/+) controls.
