# Supplementary material for: Alternating Hemiplegia of Childhood-Related Neural and Behavioural Phenotypes in Na+,K+-ATPase α3 Missense Mutant Mice
Source: PLoS One. 2013 Mar 20;8(3):e60141. doi: 10.1371/journal.pone.0060141 (PMC3603922; doi:10.1371/journal.pone.0060141)
Supplement: Table S4 — Full data for ventromedial thalamus (VMthal) functional connectivity. Data shown as the mean ± SEM of the VIP statistic as determined through PLSR analysis. Bold denotes significant functional connection with the defined seed region in given experimental group (95% CI VIP>0.80). *denotes P<0.05 significant difference in VIP statistic between genotypes (1000 random permutations). Red highlights regions showing increased and blue decreased functional connectivity with the given seed brain region in Myshkin (Myk/+) mice relative to wild-type (+/+) controls. (PDF) [file pone.0060141.s008.pdf]

Supplementary Table 4. Full data for ventromedial thalamus (VMthal) functional connectivity.

Brain Region	Wild-type (+/+)			*Myshkin* (*Myk*/+)		
	mean		SEM	mean		SEM
Caudal Motor (CMCTX)	0.46	±	0.04	0.48	±	0.06
Rostral Motor (RMCTX)	**1.50**	**±**	**0.03**	0.42*	±	0.09
Frontal Cortex (FCTX)	**1.28**	**±**	**0.04**	0.39*	±	0.05
Rostral Ventromedial Striatum (RVMStr)	**1.39**	**±**	**0.03**	**0.90**	**±**	**0.09**
Rostral Dorsolateral Striatum (RDLStr)	**1.42**	**±**	**0.02**	**1.52**	**±**	**0.04**
Rostral Dorsomedial Striatum (RDMStr)	**1.43**	**±**	**0.02**	**1.47**	**±**	**0.05**
Rostral Ventrolateral Striatum (RVLStr)	**0.99**	**±**	**0.08**	**1.44**	**±**	**0.07**
Caudal Centromedial Striatum (CCMStr)	**1.33**	**±**	**0.03**	**0.85**	**±**	**0.06**
Caudal Dorsolateral Striatum (CDLStr)	0.50	±	0.05	0.28	±	0.06
Caudal Dorsomedial Striatum (CDMStr)	**1.01**	**±**	**0.07**	0.26*	±	0.07
Caudal Ventrolateral Striatum (CVLStr)	**0.82**	**±**	**0.09**	0.67	±	0.08
Globus Pallidus (GP)	**0.99**	**±**	**0.08**	0.31*	±	0.08
Olivary Body (OB)	0.36	±	0.06	0.44	±	0.05
Auditory (AudC)	0.47	±	0.09	**1.01**	**±**	**0.05**
Substantia Nigra pars Reticulata (SNR)	0.33	±	0.03	0.46	±	0.09
CA3	0.45	±	0.08	**0.84**	**±**	**0.07**
Somatosensory (SSCTX)	**1.33**	**±**	**0.03**	0.30*	±	0.07
Ventrolateral Nucleus (VLthal)	**1.69**	**±**	**0.04**	**1.75**	**±**	**0.05**
Dorsolateral Geniculate (DLG)	0.42	±	0.06	0.82	±	0.13
CA1	**0.90**	**±**	**0.09**	0.53	±	0.06
Entorhinal (EC)	**0.89**	**±**	**0.09**	**0.86**	**±**	**0.05**
Rostral PAG (RPAG)	**0.90**	**±**	**0.06**	0.70	±	0.09
Pontine Reticular Formation (PRF)	**0.88**	**±**	**0.10**	0.76	±	0.12
Dorsolateral Entorhinal (DLECTX)	**0.97**	**±**	**0.04**	0.66	±	0.05
External Cortex of the Inferior Colliculus (ECIC)	**1.10**	**±**	**0.04**	0.54	±	0.08
Median Raphe (MR)	0.74	±	0.09	**1.34**	**±**	**0.07**
Superior Colliculus (SupC)	**1.30**	**±**	**0.03**	0.37	±	0.04
Caudal Pontine Reticular Nucleus (CPRt)	0.50	±	0.07	**1.12**	**±**	**0.06**
Superficial Grey Layer of the Superior Colliculus (SGSupC)	0.73	±	0.09	0.27	±	0.11
Caudal PAG (CPAG)	0.39	±	0.03	0.61	±	0.05
Locus Coeruleus (LC)	0.79	±	0.05	**1.64**	**±**	**0.04**
Dorsal Cortex of the Inferior Colliculus (DCIC)	**1.07**	**±**	**0.05**	0.61	±	0.09
Gigantocellular Reticular Nucleus (GRt)	**1.18**	**±**	**0.04**	**0.89**	**±**	**0.05**
Medial Vestibular nucleus (MVN)	**0.94**	**±**	**0.05**	0.53	±	0.04
Superior Cerebellar Peduncle (SupCPed)	**1.24**	**±**	**0.06**	0.62*	±	0.09
Dorsomedial PAG (DMPAG)	**1.02**	**±**	**0.06**	**0.94**	**±**	**0.06**
Medial Geniculate Nucleus (MG)	**0.84**	**±**	**0.05**	0.52	±	0.05
Paramedian Lobule (PML)	0.30	±	0.03	**1.06***	**±**	**0.03**
Red Nucleus Parvocellular (RNPv)	**0.93**	**±**	**0.10**	0.34	±	0.05
Red Nucleus Magnocellular (RNMn)	0.35	±	0.11	0.20*	±	0.02
Deep Cerebellar Nuclei (DCN)	0.33	±	0.10	0.46	±	0.01
Medial Amygdala (MeA)	0.84	±	0.07	**1.21**	**±**	**0.07**
Ventral Posterolateral Nucleus (VPLthal)	0.66	±	0.09	**2.20***	**±**	**0.03**
Ventral Posteromedial Nucleus (VPMthal)	0.68	±	0.07	**2.05***	**±**	**0.03**
Ventromedial Nucleus (VMthal)	**1.70**	**±**	**0.04**	**1.46**	**±**	**0.04**

Data shown as the mean ± SEM of the VIP statistic as determined through PLSR analysis. Bold denotes significant functional connection with the defined seed region in given experimental group (95% CI VIP>0.80). *denotes $P < 0.05$ significant difference in VIP statistic between genotypes (1000 random permutations). Red highlights regions showing increased and blue decreased functional connectivity with the given seed brain region in *Myshkin* (*Myk*/+) mice relative to wild-type (+/+) controls.
