# Supplementary material for: Alternating Hemiplegia of Childhood-Related Neural and Behavioural Phenotypes in Na+,K+-ATPase α3 Missense Mutant Mice
Source: PLoS One. 2013 Mar 20;8(3):e60141. doi: 10.1371/journal.pone.0060141 (PMC3603922; doi:10.1371/journal.pone.0060141)
Supplement: Table S5 — Full data for ventral posteromedial nucleus (VPMthal) functional connectivity. Data shown as the mean ± SEM of the VIP statistic as determined through PLSR analysis. Bold denotes significant functional connection with the defined seed region in given experimental group (95% CI VIP>0.80). *denotes P<0.05 significant difference in VIP statistic between genotypes (1000 random permutations). Red highlights regions showing increased and blue decreased functional connectivity with the given seed brain region in Myshkin (Myk/+) mice relative to wild-type (+/+) controls. (PDF) [file pone.0060141.s009.pdf]

Supplementary Table 5. Full data for ventral posteromedial nucleus (VPMthal) functional connectivity.

Brain Region	Wild-type (+/+)			*Myshkin* (*Myk*/+)		
	mean		SEM	mean		SEM
Caudal Motor (CMCTX)	0.52	±	0.05	0.51	±	0.03
Rostral Motor (RMCTX)	**1.02**	**±**	**0.06**	0.26*	±	0.06
Frontal Cortex (FCTX)	**0.95**	**±**	**0.09**	0.41	±	0.03
Rostral Ventromedial Striatum (RVMStr)	**1.00**	**±**	**0.03**	0.51*	±	0.05
Rostral Dorsolateral Striatum (RDLStr)	**0.93**	**±**	**0.04**	**0.94**	**±**	**0.03**
Rostral Dorsomedial Striatum (RDMStr)	**0.92**	**±**	**0.04**	**0.98**	**±**	**0.06**
Rostral Ventrolateral Striatum (RVLStr)	**1.02**	**±**	**0.07**	**1.03**	**±**	**0.08**
Caudal Centromedial Striatum (CCMStr)	0.75	±	0.05	0.79	±	0.06
Caudal Dorsolateral Striatum (CDLStr)	0.52	±	0.05	0.36	±	0.04
Caudal Dorsomedial Striatum (CDMStr)	**0.93**	**±**	**0.09**	0.29*	±	0.03
Caudal Ventrolateral Striatum (CVLStr)	0.72	±	0.07	0.61	±	0.05
Globus Pallidus (GP)	0.73	±	0.07	0.25*	±	0.05
Olivary Body (OB)	**1.26**	**±**	**0.14**	0.50*	±	0.08
Auditory (AudC)	0.78	±	0.10	0.81	±	0.03
Substantia Nigra pars Reticulata (SNR)	**1.48**	**±**	**0.07**	**1.06***	**±**	**0.12**
CA3	**1.37**	**±**	**0.09**	0.68*	±	0.09
Somatosensory (SSCTX)	**0.85**	**±**	**0.05**	0.60	±	0.05
Ventral Anterior Nucleus (VAthal)	**1.09**	**±**	**0.07**	**2.12***	**±**	**0.04**
Ventrolateral Nucleus (VLthal)	**0.92**	**±**	**0.09**	**1.74**	**±**	**0.03**
Dorsolateral Geniculate (DLG)	0.53	±	0.12	**1.02**	**±**	**0.08**
CA1	**1.04**	**±**	**0.07**	0.51	±	0.05
Entorhinal (EC)	**0.84**	**±**	**0.07**	0.64	±	0.06
Rostral PAG (RPAG)	**1.74**	**±**	**0.09**	**0.90**	**±**	**0.04**
Pontine Reticular Formation (PRF)	**1.23**	**±**	**0.12**	0.54*	±	0.05
Dorsolateral Entorhinal (DLECTX)	**1.19**	**±**	**0.12**	0.77	±	0.03
External Cortex of the Inferior Colliculus (ECIC)	**0.96**	**±**	**0.02**	**0.96**	**±**	**0.03**
Median Raphe (MR)	0.79	±	0.06	**1.16**	**±**	**0.04**
Superior Colliculus (SupC)	**0.87**	**±**	**0.07**	0.65	±	0.05
Caudal Pontine Reticular Nucleus (CPRt)	**1.12**	**±**	**0.08**	**0.87**	**±**	**0.06**
Superficial Grey Layer of the Superior Colliculus (SGSupC)	0.66	±	0.06	0.35	±	0.06
Caudal PAG (CPAG)	0.55	±	0.08	0.72	±	0.04
Locus Coeruleus (LC)	**1.04**	**±**	**0.08**	**1.44**	**±**	**0.02**
Dorsal Cortex of the Inferior Colliculus (DCIC)	0.65	±	0.06	0.82	±	0.11
Gigantocellular Reticular Nucleus (GRt)	**1.40**	**±**	**0.13**	**1.27**	**±**	**0.02**
Medial Vestibular nucleus (MVN)	**0.91**	**±**	**0.05**	**0.93**	**±**	**0.02**
Superior Cerebellar Peduncle (SupCPed)	**0.88**	**±**	**0.08**	**1.02**	**±**	**0.06**
Dorsomedial PAG (DMPAG)	0.78	±	0.08	**1.08**	**±**	**0.05**
Medial Geniculate Nucleus (MG)	**0.94**	**±**	**0.09**	0.70	±	0.12
Paramedian Lobule (PML)	**1.21**	**±**	**0.05**	**1.16**	**±**	**0.02**
Red Nucleus Parvocellular (RNPv)	**0.88**	**±**	**0.08**	0.40*	±	0.05
Red Nucleus Magnocellular (RNMn)	0.46	±	0.06	0.32*	±	0.05
Deep Cerebellar Nuclei (DCN)	0.54	±	0.07	0.48	±	0.08
Medial Amygdala (MeA)	**0.94**	**±**	**0.09**	0.68	±	0.06
Ventral Posterolateral Nucleus (VPLthal)	**1.20**	**±**	**0.07**	**2.12**	**±**	**0.04**
Ventromedial Thalamic Nucleus (VMthal)	**0.92**	**±**	**0.07**	**1.76***	**±**	**0.04**

Data shown as the mean ± SEM of the VIP statistic as determined through PLSR analysis. Bold denotes significant functional connection with the defined seed region in given experimental group (95% CI VIP>0.80). *denotes $P < 0.05$ significant difference in VIP statistic between genotypes (1000 random permutations). Red highlights regions showing increased and blue decreased functional connectivity with the given seed brain region in *Myshkin* (*Myk*/+) mice relative to wild-type (+/+) controls.
