# Supplementary material for: Alternating Hemiplegia of Childhood-Related Neural and Behavioural Phenotypes in Na+,K+-ATPase α3 Missense Mutant Mice
Source: PLoS One. 2013 Mar 20;8(3):e60141. doi: 10.1371/journal.pone.0060141 (PMC3603922; doi:10.1371/journal.pone.0060141)
Supplement: Table S6 — Full data for dorsomedial periaqueductal grey (DMPAG) functional connectivity. Data shown as the mean ± SEM of the VIP statistic as determined through PLSR analysis. Bold denotes significant functional connection with the defined seed region in given experimental group (95% CI VIP>0.80). *denotes P<0.05 significant difference in VIP statistic between genotypes (1000 random permutations). Red highlights regions showing increased and blue decreased functional connectivity with the given seed brain region in Myshkin (Myk/+) mice relative to wild-type (+/+) controls. (PDF) [file pone.0060141.s010.pdf]

Supplementary Table 6. Full data for dorsomedial periaqueductal grey (DMPAG) functional connectivity.

Brain Region	Wild-type (+/+)			*Myshkin* (*Myk*/+)		
	mean		SEM	mean		SEM
Caudal Motor (CMCTX)	**0.92**	**±**	**0.09**	0.57	±	0.06
Rostral Motor (RMCTX)	0.73	±	0.06	**1.42**	**±**	**0.05**
Frontal Cortex (FCTX)	0.79	±	0.07	**1.30**	**±**	**0.05**
Rostral Ventromedial Striatum (RVMStr)	0.59	±	0.06	**0.90**	**±**	**0.08**
Rostral Dorsolateral Striatum (RDLStr)	0.64	±	0.05	0.43	±	0.03
Rostral Dorsomedial Striatum (RDMStr)	0.63	±	0.05	0.32	±	0.07
Rostral Ventrolateral Striatum (RVLStr)	0.36	±	0.05	**1.05**	**±**	**0.12**
Caudal Centromedial Striatum (CCMStr)	0.59	±	0.04	0.41	±	0.04
Caudal Dorsolateral Striatum (CDLStr)	0.68	±	0.08	**0.98**	**±**	**0.06**
Caudal Dorsomedial Striatum (CDMStr)	0.61	±	0.03	0.72	±	0.05
Caudal Ventrolateral Striatum (CVLStr)	0.65	±	0.07	0.45*	±	0.08
Globus Pallidus (GP)	**0.91**	**±**	**0.10**	0.46	±	0.05
Olivary Body (OB)	0.28	±	0.05	0.81	±	0.04
Auditory (AudC)	**1.30**	**±**	**0.03**	**1.72**	**±**	**0.06**
Substantia Nigra pars Reticulata (SNR)	0.77	±	0.06	0.78	±	0.05
CA3	**1.00**	**±**	**0.06**	0.46*	±	0.16
Somatosensory (SSCTX)	0.60	±	0.05	0.70	±	0.08
Ventral Anterior Nucleus (VAthal)	**1.20**	**±**	**0.05**	**1.01**	**±**	**0.06**
Ventrolateral Nucleus (VLthal)	**1.17**	**±**	**0.05**	**1.42**	**±**	**0.08**
Dorsolateral Geniculate (DLG)	**1.74**	**±**	**0.03**	**0.94***	**±**	**0.07**
CA1	**0.90**	**±**	**0.09**	0.43*	±	0.07
Entorhinal (EC)	**1.06**	**±**	**0.10**	0.65*	±	0.07
Rostral PAG (RPAG)	**1.19**	**±**	**0.06**	0.38	±	0.12
Pontine Reticular Formation (PRF)	0.83	±	0.07	0.50	±	0.04
Dorsolateral Entorhinal (DLECTX)	**1.63**	**±**	**0.09**	**1.17**	**±**	**0.09**
External Cortex of the Inferior Colliculus (ECIC)	**0.90**	**±**	**0.06**	0.61	±	0.06
Median Raphe (MR)	0.60	±	0.07	0.71	±	0.08
Superior Colliculus (SupC)	**1.72**	**±**	**0.05**	**1.31**	**±**	**0.04**
Caudal Pontine Reticular Nucleus (CPRt)	0.49	±	0.06	0.66	±	0.05
Superficial Grey Layer of the Superior Colliculus (SGSupC)	**0.90**	**±**	**0.10**	0.53	±	0.06
Caudal PAG (CPAG)	0.81	±	0.04	0.61	±	0.08
Locus Coeruleus (LC)	0.73	±	0.03	**1.52***	**±**	**0.04**
Dorsal Cortex of the Inferior Colliculus (DCIC)	0.55	±	0.05	0.77	±	0.06
Gigantocellular Reticular Nucleus (GRt)	**0.82**	**±**	**0.06**	**1.43**	**±**	**0.08**
Medial Vestibular nucleus (MVN)	**0.96**	**±**	**0.06**	**1.33**	**±**	**0.08**
Superior Cerebellar Peduncle (SupCPed)	**1.49**	**±**	**0.07**	**0.99***	**±**	**0.07**
Medial Geniculate Nucleus (MG)	**1.46**	**±**	**0.10**	0.67*	±	0.04
Paramedian Lobule (PML)	0.31	±	0.07	0.61	±	0.06
Red Nucleus Parvocellular (RNPv)	**1.32**	**±**	**0.08**	**1.07**	**±**	**0.07**
Red Nucleus Magnocellular (RNMn)	**1.22**	**±**	**0.12**	**1.00**	**±**	**0.08**
Deep Cerebellar Nuclei (DCN)	0.80	±	0.10	**0.91**	**±**	**0.08**
Medial Amygdala (MeA)	**0.95**	**±**	**0.07**	**0.96**	**±**	**0.07**
Ventral Posterolateral Nucleus (VPLthal)	0.40	±	0.14	0.61	±	0.08
Ventral Posteromedial Nucleus (VPMthal)	0.61	±	0.06	**1.13**	**±**	**0.04**
Ventromedial Thalamic Nucleus (VMthal)	**0.93**	**±**	**0.07**	0.82	±	0.06

Data shown as the mean ± SEM of the VIP statistic as determined through PLSR analysis. Bold denotes significant functional connection with the defined seed region in given experimental group (95% CI VIP>0.80). *denotes $P < 0.05$ significant difference in VIP statistic between genotypes (1000 random permutations). Red highlights regions showing increased and blue decreased functional connectivity with the given seed brain region in *Myshkin* (*Myk*/+) mice relative to wild-type (+/+) controls.
