# Supplementary material for: Alternating Hemiplegia of Childhood-Related Neural and Behavioural Phenotypes in Na+,K+-ATPase α3 Missense Mutant Mice
Source: PLoS One. 2013 Mar 20;8(3):e60141. doi: 10.1371/journal.pone.0060141 (PMC3603922; doi:10.1371/journal.pone.0060141)
Supplement: Table S7 — Full data for rostral periaqueductal grey (RPAG) functional connectivity. Data shown as the mean ± SEM of the VIP statistic as determined through PLSR analysis. Bold denotes significant functional connection with the defined seed region in given experimental group (95% CI VIP>0.80). *denotes P<0.05 significant difference in VIP statistic between genotypes (1000 random permutations). Red highlights regions showing increased and blue decreased functional connectivity with the given seed brain region in Myshkin (Myk/+) mice relative to wild-type (+/+) controls. (PDF) [file pone.0060141.s011.pdf]

Supplementary Table 7. Full data for rostral periaqueductal grey (RPAG) functional connectivity.

Brain Region	Wild-type (+/+)			*Myshkin* (*Myk*/+)		
	mean		SEM	mean		SEM
Caudal Motor (CMCTX)	0.76	±	0.05	0.46	±	0.06
Rostral Motor (RMCTX)	**1.00**	**±**	**0.05**	0.59	±	0.05
Frontal Cortex (FCTX)	**0.93**	**±**	**0.05**	0.81	±	0.10
Rostral Ventromedial Striatum (RVMStr)	**0.90**	**±**	**0.07**	0.61	±	0.04
Rostral Dorsolateral Striatum (RDLStr)	**0.88**	**±**	**0.08**	0.31*	±	0.03
Rostral Dorsomedial Striatum (RDMStr)	**0.88**	**±**	**0.07**	0.55	±	0.07
Rostral Ventrolateral Striatum (RVLStr)	0.63	±	0.05	**1.26**	**±**	**0.09**
Caudal Centromedial Striatum (CCMStr)	0.74	±	0.06	**1.58***	**±**	**0.04**
Caudal Dorsolateral Striatum (CDLStr)	0.71	±	0.04	**1.02**	**±**	**0.09**
Caudal Dorsomedial Striatum (CDMStr)	0.67	±	0.03	**1.04**	**±**	**0.07**
Caudal Ventrolateral Striatum (CVLStr)	0.68	±	0.09	**1.30**	**±**	**0.07**
Globus Pallidus (GP)	**1.07**	**±**	**0.03**	0.42*	±	0.06
Olivary Body (OB)	**0.95**	**±**	**0.08**	**1.04**	**±**	**0.07**
Auditory (AudC)	0.73	±	0.06	0.57*	±	0.08
Substantia Nigra pars Reticulata (SNR)	**1.82**	**±**	**0.06**	0.83*	±	0.08
CA3	**1.08**	**±**	**0.06**	**1.19**	**±**	**0.13**
Somatosensory (SSCTX)	**0.76**	**±**	**0.06**	**0.76**	**±**	**0.07**
Ventral Anterior Nucleus (VAthal)	**1.12**	**±**	**0.05**	**0.86**	**±**	**0.11**
Ventrolateral Nucleus (VLthal)	**1.10**	**±**	**0.05**	0.41*	±	0.13
Dorsolateral Geniculate (DLG)	**0.89**	**±**	**0.08**	0.53*	±	0.09
CA1	0.70	±	0.07	0.36*	±	0.05
Entorhinal (EC)	0.64	±	0.03	0.75	±	0.06
Pontine Reticular Formation (PRF)	**1.55**	**±**	**0.05**	0.49	±	0.09
Dorsolateral Entorhinal (DLECTX)	**1.11**	**±**	**0.05**	**1.32**	**±**	**0.04**
External Cortex of the Inferior Colliculus (ECIC)	0.82	±	0.06	0.47	±	0.09
Median Raphe (MR)	0.65	±	0.07	**0.86**	**±**	**0.08**
Superior Colliculus (SupC)	**1.34**	**±**	**0.06**	**1.96***	**±**	**0.06**
Caudal Pontine Reticular Nucleus (CPRt)	0.62	±	0.04	0.72	±	0.09
Superficial Grey Layer of the Superior Colliculus (SGSupC)	0.73	±	0.08	**2.02***	**±**	**0.08**
Caudal PAG (CPAG)	0.43	±	0.07	**0.91**	**±**	**0.09**
Locus Coeruleus (LC)	0.59	±	0.03	**1.33**	**±**	**0.08**
Dorsal Cortex of the Inferior Colliculus (DCIC)	0.64	±	0.04	0.81	±	0.09
Gigantocellular Reticular Nucleus (GRt)	**1.74**	**±**	**0.04**	0.43	±	0.10
Medial Vestibular nucleus (MVN)	**1.49**	**±**	**0.04**	0.41	±	0.08
Superior Cerebellar Peduncle (SupCPed)	**0.82**	**±**	**0.03**	0.45	±	0.11
Dorsomedial PAG (DMPAG)	**1.16**	**±**	**0.06**	0.40*	±	0.13
Medial Geniculate Nucleus (MG)	**0.84**	**±**	**0.06**	**1.58**	**±**	**0.07**
Paramedian Lobule (PML)	0.58	±	0.06	**1.54**	**±**	**0.08**
Red Nucleus Parvocellular (RNPv)	**1.15**	**±**	**0.07**	**1.74**	**±**	**0.06**
Red Nucleus Magnocellular (RNMn)	0.76	±	0.03	**1.31**	**±**	**0.06**
Deep Cerebellar Nuclei (DCN)	0.48	±	0.09	0.75	±	0.06
Medial Amygdala (MeA)	0.76	±	0.06	0.45*	±	0.03
Ventral Posterolateral Nucleus (VPLthal)	0.57	±	0.09	**1.05**	**±**	**0.13**
Ventral Posteromedial Nucleus (VPMthal)	**1.57**	**±**	**0.12**	**1.01**	**±**	**0.05**
Ventromedial Thalamic Nucleus (VMthal)	**0.97**	**±**	**0.07**	0.57	±	0.12

Data shown as the mean ± SEM of the VIP statistic as determined through PLSR analysis. Bold denotes significant functional connection with the defined seed region in given experimental group (95% CI VIP>0.80). *denotes $P < 0.05$ significant difference in VIP statistic between genotypes (1000 random permutations). Red highlights regions showing increased and blue decreased functional connectivity with the given seed brain region in *Myshkin* (*Myk*/+) mice relative to wild-type (+/+) controls.
