# Supplementary material for: Alternating Hemiplegia of Childhood-Related Neural and Behavioural Phenotypes in Na+,K+-ATPase α3 Missense Mutant Mice
Source: PLoS One. 2013 Mar 20;8(3):e60141. doi: 10.1371/journal.pone.0060141 (PMC3603922; doi:10.1371/journal.pone.0060141)
Supplement: Table S8 — Full data for caudal motor cortex (CMCTX) functional connectivity. Data shown as the mean ± SEM of the VIP statistic as determined through PLSR analysis. Bold denotes significant functional connection with the defined seed region in given experimental group (95% CI VIP>0.80). *denotes P<0.05 significant difference in VIP statistic between genotypes (1000 random permutations). Red highlights regions showing increased and blue decreased functional connectivity with the given seed brain region in Myshkin (Myk/+) mice relative to wild-type (+/+) controls. (PDF) [file pone.0060141.s012.pdf]

Supplementary Table 8. Full data for caudal motor cortex (CMCTX) functional connectivity.

Brain Region	Wild-type (+/+)			*Myshkin* (*Myk*/+)		
	mean		SEM	mean		SEM
Rostral Motor (RMCTX)	0.71	±	0.10	**1.46**	**±**	**0.03**
Frontal Cortex (FCTX)	**0.89**	**±**	**0.11**	0.52	±	0.02
Rostral Ventromedial Striatum (RVMStr)	0.56	±	0.11	0.73	±	0.09
Rostral Dorsolateral Striatum (RDLStr)	0.58	±	0.09	0.43	±	0.04
Rostral Dorsomedial Striatum (RDMStr)	0.58	±	0.09	0.61	±	0.08
Rostral Ventrolateral Striatum (RVLStr)	0.48	±	0.14	0.53	±	0.04
Caudal Centromedial Striatum (CCMStr)	**1.26**	**±**	**0.07**	**1.72**	**±**	**0.04**
Caudal Dorsolateral Striatum (CDLStr)	**1.99**	**±**	**0.03**	**2.10**	**±**	**0.01**
Caudal Dorsomedial Striatum (CDMStr)	**1.39**	**±**	**0.07**	**2.15**	**±**	**0.02**
Caudal Ventrolateral Striatum (CVLStr)	**1.55**	**±**	**0.03**	**1.35***	**±**	**0.05**
Globus Pallidus (GP)	0.75	±	0.04	**2.07***	**±**	**0.03**
Olivary Body (OB)	0.63	±	0.08	**1.00**	**±**	**0.05**
Auditory (AudC)	**1.34**	**±**	**0.05**	0.31*	±	0.11
Substantia Nigra pars Reticulata (SNR)	**0.98**	**±**	**0.06**	0.44*	±	0.05
CA3	0.59	±	0.06	**1.25**	**±**	**0.05**
Somatosensory (SSCTX)	**0.92**	**±**	**0.09**	**0.91**	**±**	**0.06**
Ventral Anterior Nucleus (VAthal)	0.55	±	0.06	0.50	±	0.08
Ventrolateral Nucleus (VLthal)	0.57	±	0.06	0.62	±	0.08
Dorsolateral Geniculate (DLG)	**1.35**	**±**	**0.07**	**1.70**	**±**	**0.06**
CA1	**1.20**	**±**	**0.08**	**0.95**	**±**	**0.08**
Entorhinal (EC)	0.84	±	0.08	0.50	±	0.13
Rostral Periaqueductal Grey (RPAG)	0.59	±	0.05	0.36	±	0.05
Pontine Reticular Formation (PRF)	0.39	±	0.04	**1.08***	**±**	**0.10**
Dorsolateral Entorhinal (DLECTX)	**0.87**	**±**	**0.08**	0.43	±	0.02
External Cortex of the Inferior Colliculus (ECIC)	0.62	±	0.09	**0.98**	**±**	**0.13**
Median Raphe (MR)	**1.75**	**±**	**0.03**	**1.10**	**±**	**0.08**
Superior Colliculus (SupC)	0.52	±	0.05	**1.09***	**±**	**0.08**
Caudal Pontine Reticular Nucleus (CPRt)	**1.72**	**±**	**0.07**	0.39*	±	0.07
Superficial Grey Layer of the Superior Colliculus (SGSupC)	0.82	±	0.06	**1.03**	**±**	**0.07**
Caudal PAG (CPAG)	**1.11**	**±**	**0.09**	**0.93**	**±**	**0.12**
Locus Coeruleus (LC)	**1.53**	**±**	**0.04**	0.48*	±	0.10
Dorsal Cortex of the Inferior Colliculus (DCIC)	**1.65**	**±**	**0.03**	0.60	±	0.11
Gigantocellular Reticular Nucleus (GRt)	0.51	±	0.04	0.65	±	0.06
Medial Vestibular nucleus (MVN)	0.49	±	0.06	0.69	±	0.04
Superior Cerebellar Peduncle (SupCPed)	0.41	±	0.07	0.44	±	0.11
Dorsomedial PAG (DMPAG)	**0.86**	**±**	**0.08**	0.48	±	0.06
Medial Geniculate Nucleus (MG)	0.38	±	0.03	0.70	±	0.10
Paramedian Lobule (PML)	**1.37**	**±**	**0.11**	0.46*	±	0.03
Red Nucleus Parvocellular (RNPv)	0.45	±	0.05	0.44	±	0.03
Red Nucleus Magnocellular (RNMn)	0.59	±	0.09	0.45	±	0.07
Deep Cerebellar Nuclei (DCN)	**1.31**	**±**	**0.10**	**1.45**	**±**	**0.07**
Medial Amygdala (MeA)	**0.92**	**±**	**0.10**	**0.95**	**±**	**0.07**
Ventral Posterolateral Nucleus (VPLthal)	0.35	±	0.09	0.44	±	0.09
Ventral Posteromedial Nucleus (VPMthal)	0.37	±	0.04	0.50	±	0.05
Ventromedial Thalamic Nucleus (VMthal)	0.68	±	0.07	0.35	±	0.06

Data shown as the mean ± SEM of the VIP statistic as determined through PLSR analysis. Bold denotes significant functional connection with the defined seed region in given experimental group (95% CI VIP>0.80). *denotes $P < 0.05$ significant difference in VIP statistic between genotypes (1000 random permutations). Red highlights regions showing increased and blue decreased functional connectivity with the given seed brain region in *Myshkin* (*Myk*/+) mice relative to wild-type (+/+) controls.
